# How Do the Virulence Factors of *Shigella* Work Together to Cause Disease?

**DOI:** 10.3389/fcimb.2017.00064

**Published:** 2017-03-24

**Authors:** Emily Mattock, Ariel J. Blocker

**Affiliations:** Faculty of Biomedical Sciences, Schools of Cellular and Molecular Medicine and Biochemistry, University of BristolBristol, UK

**Keywords:** Shigellosis, *Shigella*, bacterial pathogenesis, type III secretion system, virulence effectors

## Abstract

*Shigella* is the major cause of bacillary dysentery world-wide. It is divided into four species, named *S. flexneri, S. sonnei, S. dysenteriae*, and *S. boydii*, which are distinct genomically and in their ability to cause disease. Shigellosis, the clinical presentation of *Shigella* infection, is characterized by watery diarrhea, abdominal cramps, and fever. *Shigella*'s ability to cause disease has been attributed to virulence factors, which are encoded on chromosomal pathogenicity islands and the virulence plasmid. However, information on these virulence factors is not often brought together to create a detailed picture of infection, and how this translates into shigellosis symptoms. Firstly, *Shigella* secretes virulence factors that induce severe inflammation and mediate enterotoxic effects on the colon, producing the classic watery diarrhea seen early in infection. Secondly, *Shigella* injects virulence effectors into epithelial cells via its Type III Secretion System to subvert the host cell structure and function. This allows invasion of epithelial cells, establishing a replicative niche, and causes erratic destruction of the colonic epithelium. Thirdly, *Shigella* produces effectors to down-regulate inflammation and the innate immune response. This promotes infection and limits the adaptive immune response, causing the host to remain partially susceptible to re-infection. Combinations of these virulence factors may contribute to the different symptoms and infection capabilities of the diverse *Shigella* species, in addition to distinct transmission patterns. Further investigation of the dominant species causing disease, using whole-genome sequencing and genotyping, will allow comparison and identification of crucial virulence factors and may contribute to the production of a pan-*Shigella* vaccine.

## Introduction

*Shigella* was recognized as the causative agent of bacillary dysentery in 1897 by Kiyoshi Shiga. He determined that it was a Gram negative bacillus, which was capable of fermenting dextrose, but was indole-reaction negative and incapable of producing acid from mannitol (Trofa et al., 1999). *Shigella* is a non-sporulating, facultative anaerobe. *Shigella* is also a primate-restricted pathogen, which differentiates it from the other members of the Enterobacteriaceae family in which it is classified.

The *Shigella* genus is divided into four species: *Shigella dysenteriae* (serogroup A, 15 serotypes), *Shigella flexneri* (serogroup B, 19 serotypes), *Shigella boydii* (serogroup C, 20 serotypes), and *Shigella sonnei* (serogroup D, 1 serotype). These are divided into multiple serotypes dependent on O-antigen and biochemical differences. Different species are linked to disease in varying geographical locations. *S. dysenteriae* causes severe epidemic disease in less developed countries, *S. flexneri* causes disease in developing countries, *S. boydii* is confined to the Indian subcontinent, and *S. sonnei* occurs in both transitional and developed countries (Levine et al., 2013).

**Figure 1:**
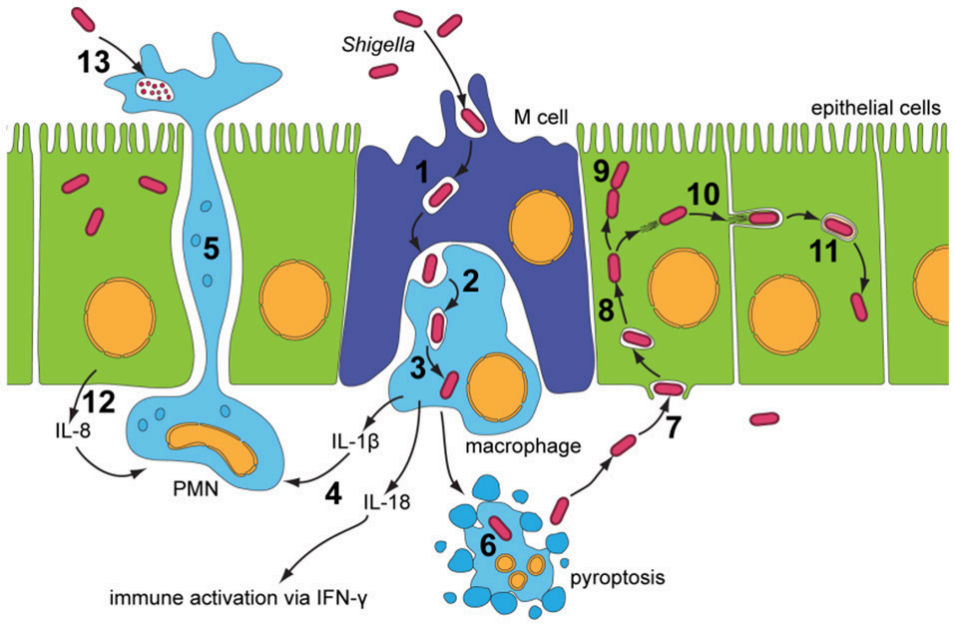
**Infectious cycle of *Shigella* (Roerich-Doenitz, 2013) modified from Schroeder and Hilbi (2008)**. Entry into the colonic epithelium is mediated in two ways: M-cell membrane ruffling, and epithelial barrier destabilization. Entry via M-cells is achieved through membrane ruffling (**1**), and the bacillus is then transported to the M-cell pocket, where it is endocytosed by resident macrophages (**2**). Epithelial barrier destruction is mediated by pro-inflammatory (IL-1) and chemotaxic cytokines (IL-8). IL-8 produced by neighboring epithelial cells recruits PMN leukocytes (**12**), which travel from the basolateral to the apical colonic epithelium, destabilizing the junctions between the epithelial cells and allowing further invasion of *Shigella* (**5**). Induction of pyroptotic macrophage death occurs after *Shigella* escape from the phagocytic vacuole (**3** and **6**). Caspase-1, when activated, cleaves and activates IL-1β and IL-18, leading to the release of these pro-inflammatory cytokines (**4**) (Jennison and Verma, 2004). Uptake of *Shigella* is a macropinocytic process at the basolateral membrane of epithelial cells (**7**). Stimulation of Rho-family GTPases triggers actin polymerisation and then depolymerization, forming filopodial and lamellipodial extensions of the epithelial membrane, leading to engulfment of the bacilli. Lysis of the macropinocytic vacuole allows *Shigella* to gain access to the epithelial cytoplasm, where it rapidly multiplies, escapes autophagy and fragments the Golgi (**8** and **9**). Exploitation of the epithelial actin assembly machinery allows *Shigella* to move both intra- and intercellularly (**10**). Protrusions mediated by bacilli are actively endocytosed by the clathrin-mediated endocytic pathways at intercellular junctions, and the double membrane vacuole is lysed to give *Shigella* access to the neighboring cells cytoplasm (**11**). PMN leukocytes eventually eliminate *Shigella* infection from the colonic epithelium (**13**).

Shigellosis is the clinical presentation of *Shigella* infection. Disease is transmitted through the fecal-oral route, with an infectious dose of only 10–100 organisms (Levine et al., 2013). After 1–4 days, infection is acute, non-systemic and enterically invasive, leading to destruction of the colonic epithelium (detailed in Figure 1). Damage along the colonic epithelial is dramatic but erratic, and leads to the main clinical symptom of diarrhea, containing blood and sometimes mucus, which may be accompanied by abdominal cramps and fever. Further complications, depending on the infecting *Shigella* species and host HLA subtype, include Haemolytic-Uremic Syndrome (HUS) and Post-Reactive Arthritis (WHO, 2005). HUS occurs in 2–7% of *S. dysenteriae* type 1 infections, whereby the Shiga toxin harbored by this species attaches to the endothelium and activates platelets, which adhere to the endothelium and occlude blood vessels leading microangiopathic haemolysis of red blood cells as they squeeze through the restricted blood vessel lumen (O'Loughlin and Robins-Browne, 2001). Symptoms include acute renal failure, thrombocytopenia, micro-angiopathic haemolytic anemia, with a 35% fatality rate (Mayer et al., 2012). Post-reactive arthritis is another complication of *Shigella* infection, occurring in 2% of cases, and is characterized by painful joints, painful urination, and irritation of eyes, with chronic arthritis lasting from months to years.

**Figure 2:**
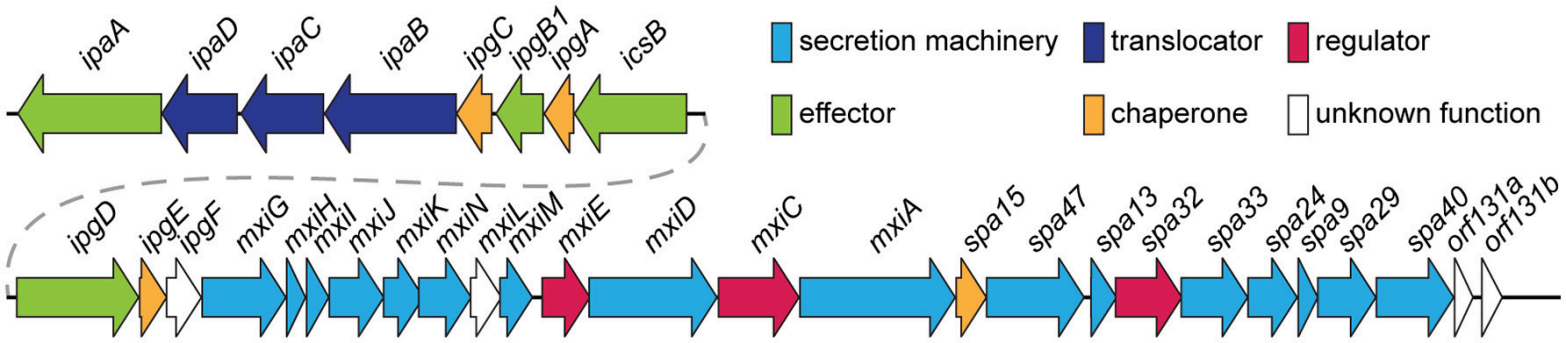
**Genomic organization of the entry region on plasmid pWR100 (Roerich-Doenitz, 2013)**. Genes are clustered in two operons, the *ipa*/*ipg* and the *mxi*/*spa* operon. They are colored in the legend according to their protein class, some of which, such as T3SS *effectors*, are detailed in the text. *Secretion machinery* refers to the components that build the T3SS. *Translocators* are components of the translocon, a pore inserted into the host membrane to allow effector translocation and *chaperones* are components that stabilize individual effectors prior to secretion from the bacterium. *Regulators* modulate T3SS expression and function but they are largely beyond the scope of this review. This figure was modified from the virulence plasmid map by Buchrieser et al. (2000).

Comparison of the main subtypes of these species by Yang et al. (2005) indicates that each *Shigella* species contains a single circular chromosome and a virulence plasmid. The virulence plasmid has been thoroughly researched in relation to pathogenesis, and the majority of the important virulence factors involved in the *Shigella* life-cycle are localized to a 30 kb region termed the “entry region” (Figure 2). This region contains the *mxi*-*spa* locus, which encodes the Type III Secretion System (T3SS), and *ipa* and *ipg* genes, which are essential for invasion of epithelial cells and initiation of *Shigella* infection. In addition to the virulence plasmid, distinct regions within the *Shigella* chromosome have also been shown to contribute to infection. These are termed “pathogenicity islands” (PAI) (Table 1), which are unstable transferable elements that can be found in a variety of combinations depending on the *Shigella* species and subtype (Yang et al., 2005). A combination of both chromosomal virulence factors and plasmid virulence factors mediate the *Shigella* life cycle that leads to destruction of the colonic epithelium and disease symptoms.

**Table 1:** **Genes and protein functions involved in virulence on the *Shigella* chromosome**.

**PAI**	**Gene(s)**	**Protein function**	**References**
SHI-1	*sigA*	Putative enterotoxin	Al-Hasani et al., 2009
	*pic*	Intestinal colonization	Navarro-Garcia et al., 2010
	*set1A, set1B*	ShET1 enterotoxin	Fasano et al., 1997
SHI-2	*iucA–D*	Siderophore, complexes with iron	Vokes et al., 1999
	*iutA*	Bacterial receptor for iron-siderophore complex	Vokes et al., 1999
	*shiA–G*	Novel ORFS, ShiA involved in reduction in host inflammatory response	Ingersoll et al., 2003
SHI-3	*iucA–D*	Siderophore, complexes with iron	Purdy and Payne, 2001
	*iutA*	Bacterial receptor for iron-siderophore complex	Purdy and Payne, 2001
SHI-O	*gtrA, gtrB, gtr*	Serotype conversion and O-antigen modification	Allison and Verma, 2000
Stx-phage P27	*stxAB*	Shiga toxin	Yang et al., 2005

**Figure 3:**
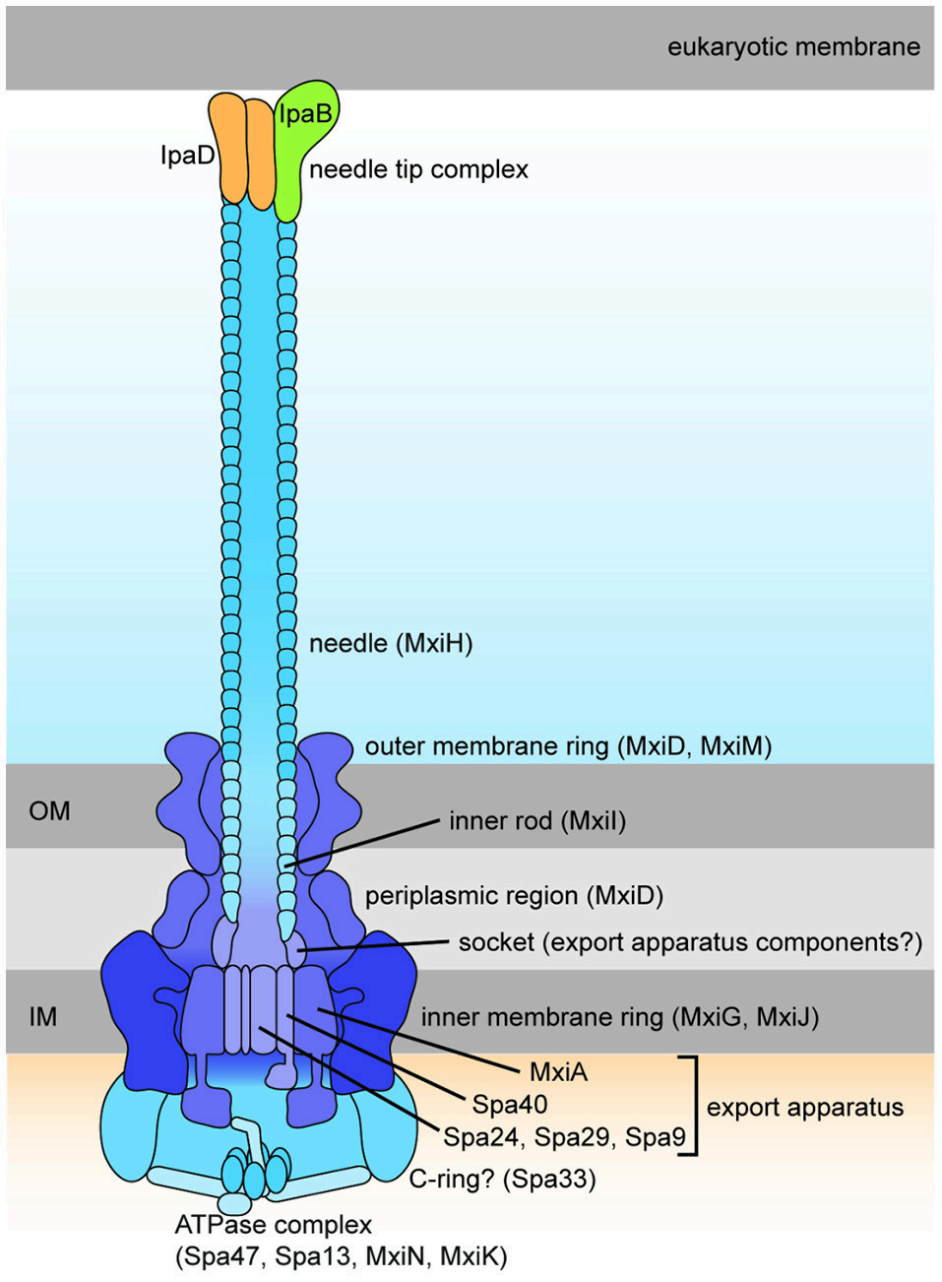
**Schematic drawing of the *Shigella* Type III Secretion System (Roerich-Doenitz, 2013)**. The architecture of *Shigella* T3SS is based on the virulence plasmid *mxi*-*spa* locus (Figure 2), which encodes proteins that produce the T3SS apparatus, a structure composed of a 60 nm hollow extracellular needle, a transmembrane domain, and a cytoplasmic bulb (Blocker et al., 1999).

The T3SS harbored by *Shigella* is pivotal to infection, delivering from the bacterial cytoplasm into the host cell effectors that play a role in cellular invasion, manipulation, and apoptosis (Parsot, 2009). At 37°C the T3SS components are assembled (Figure 3) but secretion of effectors is prevented until the T3SS is activated by contact with the host cell (Veenendaal et al., 2007). These effectors can be classified dependent on the timing of gene expression as either early, middle, or late effectors, and unless stated otherwise, all of the effectors discussed below are encoded on the virulence plasmid and secreted in a T3SS-dependent manner (Table 2).

**Table 2:** **Effectors secreted in a T3SS-dependent manner**.

**Effectors**	**Early/Middle/Late**	**Enzyme activity**	**Function**	**References**
IcsB	Early		Inhibits autophagy	Allaoui et al., 1992; Ambrosi et al., 2014; Baxt and Goldberg, 2014; Huang and Brumell, 2014; Campbell-Valois et al., 2015
IpaA	Early		Actin depolymerization	Tran Van Nhieu et al., 1997; Bourdet-Sicard et al., 1999; Izard et al., 2006; Ramarao et al., 2007
IpaB	Early		Translocon pore formation	High et al., 1992; Thirumalai et al., 1997; Page et al., 1999; Skoudy et al., 2000; Lafont et al., 2002; Yang et al., 2013; Suzuki et al., 2014b
IpaC	Early		Translocon pore formation; Actin polymerization; Docking and effector induction.	Bârzu et al., 1997; Tran Van Nhieu et al., 1999; Osiecki et al., 2001; Terry et al., 2008; Mounier et al., 2009; Du et al., 2016; Russo et al., 2016
IpaD	Early		Activation of T3SS	Blocker et al., 1999; Arizmendi et al., 2016
IpaH0722		E3 ubiquitin ligase	Ubiquitinates TRAF2	Ashida et al., 2013
IpaH7.8	Late	E3 ubiquitin ligase	Ubiquitinates glomulin	Suzuki et al., 2014a
IpaH9.8	Late	E3 ubiquitin ligase	Ubiquitinates U2AF^35^ and NEMO/IKKγ	Okuda et al., 2005; Ashida et al., 2010
IpaJ		Cysteine protease	Cleaves ARF1-GTP	Burnaevskiy et al., 2015; Dobbs et al., 2015
IpgB1	Early	GEF	Activates Rac1 and Cdc42	Ohya et al., 2005; Hachani et al., 2007; Huang et al., 2009
IpgB2	Early	GEF	Activates RhoA	Hachani et al., 2007; Huang et al., 2009; Klink et al., 2010
IpgD	Early	Inositol 4-phosphatase	Converts PtdIns(4,5)P_2_ to PtdIns(5)P	Niebuhr et al., 2000; Mayo and Donner, 2001; Mellouk et al., 2014; Garza-Mayers et al., 2015
OspB	Middle		Phosphorylation of ERK and p38 MAPK	Zurawski et al., 2009; Ambrosi et al., 2014; Lu et al., 2015
OspC1	Middle		Phosphorylation of ERK	Zurawski et al., 2006
OspC3	Early		Binds p19	Kobayashi et al., 2013
OspD3	Late		Enterotoxic activity	Farfán et al., 2011; Faherty et al., 2016
OspE1/2	Late		Adhesin Interacts with ILK	Miura et al., 2006; Kim et al., 2009; Faherty et al., 2012a
OspF	Middle	Phosphothr-eonine lyase	Dephosphorylates MAPKs	Arbibe et al., 2007; Li et al., 2007; Zhu et al., 2007
OspG	Late	Kinase	Inhibits SCF^β−TrCP^ ubiquitinating IκBα	Kim et al., 2005; Zhou et al., 2013; Grishin et al., 2014; Pruneda et al., 2014
OspI		Glutamine deamidase	Deamidates UBC13	Sanada et al., 2012; Nishide et al., 2013
OspZ_1–188_			Phosphorylation of ERK	Newton et al., 2010
OspZ			Blocks p65 nuclear translocation	Zurawski et al., 2008; Nadler et al., 2010
VirA	Middle	GAP	Inactivates Rab proteins	Germane et al., 2008; Dong et al., 2012; Campbell-Valois et al., 2015

Dysentery, the main clinical symptom of shigellosis, is due to the infectious cycle of *Shigella* and its ability to penetrate and colonize the colonic epithelium, leading to loss of barrier function and inflammation (Jennison and Verma, 2004). This initial inflammation (Figure 1) is paramount for efficient infection. However, *Shigella* must also overcome this innate immune response and dampen inflammation in order to establish infection, especially in the epithelial cell niche. *Shigella* diminishes the inflammatory response by delivering effectors to inhibit the NFκB and MAPK signaling pathways and epigenetically regulate the repression of pro-inflammatory cytokines such as IL-8 (Ashida et al., 2011). In addition, *Shigella* is capable of downregulating production of antimicrobial peptides, including human β-defensin hBD-3, and chemokines, such as CCL20, leading to defective dendritic cell recruitment (Sperandio et al., 2008). This allows for increased replication, efficient infection of neighboring cells, and evasion of the immune response (Figure 1). Eventually, however, the initial inflammatory response which allows for efficient infection consequently leads to *Shigella* clearance. Polymorphonuclear leukocytes (PMN), such as neutrophils, eliminate the infection within 5–7 days in healthy individuals (Figure 1).

*Shigella* has evolved to successfully re-infect its host and probably subverts the production of efficient immunological memory to do so. After infection, seroconversion produces protective antibodies against *Shigella* lipopolysaccharide (LPS), however the antibodies produced are serogroup specific. The diversity of *Shigella* LPS serotypes means that protection against re-infection is limited to homologous disease, and these LPS-specific antibodies are also short lasting (Cohen et al., 1991). Progressive acquisition of pan-species immunological memory occurs after many infections, and is probably achieved through recognition of protein-based specific antigens, such as the “invasion plasmid antigens” (Ipa) proteins. These virulence plasmid-encoded antigens are therefore important targets for vaccine development (Levine et al., 2013). The adaptive immune response, including T- and B-lymphocytes, takes 4–7 days to begin working efficiently, which coincides with resolution of *Shigella* infection in healthy individuals. *Shigella* prevents dendritic cell recruitment by downregulating the main chemoattractant CCL20, as previously described, and also mediates dendritic cell apoptosis (Kim et al., 2008). Dendritic cells are a key link between the innate and adaptive immune response, and the inhibition of their function consequently interferes with the T_H_1-T_H_2-T_H_17 transition required for an efficient adaptive immune response (Sperandio et al., 2008). Therefore, it is possible that prevention of an efficient adaptive immune response is achieved via a combination of innate immune system modulation and subversion of immunological memory production.

The individual virulence factors of *Shigella* have been compiled and reviewed previously. Here we aim to understand how they collaborate to cause acute enteric destruction, leading to the clinical manifestation of shigellosis. We will analyse the available primary data for the function of *Shigella* effector proteins and their effect on host cells, and then discuss how these are co-ordinated in time and space to create a detailed picture of *Shigella* infection, how it leads to disease and manipulates the immune response.

## Analysis

### Epithelial barrier destabilization and inflammation

#### OspB: promotes PMN migration, inflammation, and cell proliferation

Like most effectors, OspB is found in the four *Shigella* species, and has homolog in *Salmonella* species (Zurawski et al., 2009). Although its biochemical function is unknown, it is thought that OspB plays a role in the activation of extracellular-signal-regulated kinases (ERK) and p38 MAPK pathways, resulting in phosphorylation of phospholipase A2 and the generation of eicosanoids. OspB is capable of nuclear localization for activation of MAPK signaling pathways. This contributes to inflammation and PMN migration, possibly inducing hepoxilin A3, an arachidonic acid derivative, and apical secretion of IL-8, a PMN chemoattractant (Ambrosi et al., 2014). An *ospB*^−^ mutant had a 60% decrease in PMN migration and a 30% decrease in ERK1/2 activation 90 min post-infection when compared to wild-type *Shigella* (Zurawski et al., 2009). Furthermore, Ambrosi et al. (2014) showed that an *ospB*^−^ knockout displayed significantly reduced onset and severity of symptoms in the guinea pig keratoconjunctivitis model of infection (Sereny test). However, OspB also activates the master regulator of cell growth mTOR via a direct interaction with the cellular scaffold protein IQGAP1, which also interacts with mTOR activators ERK1/2. This seems to restricts the spread of *S. flexneri* in cell monolayers, possibly by enhancing cell proliferation in infected foci (Lu et al., 2015).

#### OspC1: promotes PMN migration and inflammation

OspC1 is part of the *ospC* family. There is 96% identity between *ospC2, ospC3*, and *ospC4*, but only 74% identity between these three *ospC* genes and *ospC1* (Buchrieser et al., 2000). This level of similarity may indicate redundancy. However, *ospC4* is a pseudogene and different functions have been identified for OspC1 and OspC3 (discussed later). Tagged OspC1 is found throughout the host cytoplasm, localizing primarily to the nucleus (Zurawski et al., 2006). An *ospC1*^−^ knockout showed a significant decrease in the amount of neutrophil recruitment to the epithelial cells in PMN migration assays, which was restored to wild-type levels on complementation with a plasmid expressing *ospC1*. Zurawski et al. (2006) showed that this increase in PMN migration correlated with increase in the phosphorylation of ERK1/2 pathways mediated by OspC1. An *ospC1*^−^ knockout showed a decrease in phosphorylation of ERK1/2 compared to wild-type levels but no reduction in IL-8 secretion. OspC1 plays a role in *Shigella* virulence *in vivo* as an *ospC1*^−^ knockout had reduced amounts of swelling and inflammation in the Sereny test, with clearance of infection after 2 days (Zurawski et al., 2006).

#### OspZ: promotes PMN migration and inflammation

In *S. flexneri 2a*, an *ospZ*^−^ knockout has no effect on the Sereny test. However, an *ospZ*^−^ knockout caused a significant decrease in PMN migration. The knockout also had 63 and 53% ERK1/2 phosphorylation and NFκB activation, respectively, when compared to wild-type *S. flexneri* (Zurawski et al., 2008). OspZ therefore plays a role in the migration of PMN leukocytes across the epithelial barrier. However, Newton et al. (2010) discovered that *S. flexneri* species, excluding *S. flexneri* serotype 6, contain a stop codon at amino acid 188, forming a truncated protein lacking an IDSYMK motif at position 209. The full length OspZ proteins in the remaining *Shigella* species were found to have an immunosuppressive function through prevention of NFκB activation. Finally, an OspZ homolog, NleE, is found in enteropathogenic *Escherichia coli* (EPEC), and both NleE and OspZ can substitute for each other (Zurawski et al., 2008).

#### Serine protease autotransporters of enterobacteriaceae

Serine Protease Autotransporters of Enterobacteriaceae (SPATEs) are a family of proteases which catalyse their own secretion via the Type V secretion pathway. *Shigella* has three known SPATEs, not all of which are found in each species. Their secretion is thermoregulated (37°C) and pH-dependent (Dautin, 2010). They have different proposed activities relevant to intestinal penetration: induction of mucin secretion and cleavage (Pic), destabilization of focal adhesions via cleavage of fodrin (SigA), and, through unknown targets, enterotoxicity, fluid accumulation and epithelial desquamation (SigA and SepA) (Table 3).

**Table 3:** **SPATEs harbored by *Shigella* and their function in infection**.

**SPATE**	**Gene location**	**Putative function**	**Role in infection**	**References**
Pic	SHI-1 (opposite *set1AB*)	Cleavage of mucin	Penetrate colonic mucus layer to access epithelium	Gutierrez-Jimenez et al., 2008
		Mucin secretagogue	Mucus-containing dysentery in shigellosis	Navarro-Garcia et al., 2010
SigA	SHI-1	Cytopathic activity	Cleavage of fodrin to destabilize links between actin cytoskeleton and membrane proteins, detachment of focal adhesions	Canizalez-Roman and Navarro-García, 2003; Al-Hasani et al., 2009
		Enterotoxic activity	Fluid accumulation	Al-Hasani et al., 2000
SepA	Virulence plasmid	Enterotoxic activity	Fluid accumulation	Benjelloun-Touimi et al., 1995
		Epithelial desquamation	Disease progression	Coron et al., 2009

#### *Shigella* enterotoxin 1 and *Shigella* enterotoxin 2: enterotoxic activity in the jejunum

*Shigella* enterotoxin 1 (ShET1) and *Shigella* enterotoxin 2 (ShET2) are virulence determinants proposed to mediate early fluid secretion in the jejunum to establish infection in the colon and produce to the characteristic watery diarrhea seen early in shigellosis. The shared name is due to their similar properties as enterotoxins, as there is no homology between ShET1 and ShET2.

ShET1 is encoded by *set1A* and *set1B* genes on the *Shigella* chromosome as part of the SHI-1 PAI, and only present in *S. flexneri* 2a isolates (Vargas et al., 1999) (Yavzori et al., 2002). The two subunits are proposed to form a holo-AB-type toxin complex in an A1-B5 configuration, producing a 55 kDa complex (Fasano et al., 1995). The holotoxin may follow a secretion mechanism similar to that of the cholera holotoxin, via the Sec pathway and Type II secretion. When ion transport across a cultured epithelium was measured in an Using chamber, a *set1AB*^−^ knockout had 60% lower I_sc_ (short circuit current) in comparison to wild-type strains (Faherty et al., 2012b). The effect on I_sc_ of ShET1 was also dose-dependent, and washout of ShET1 produced no change in I_sc_, indicating irreversible binding of ShET1 to epithelial receptors (Fasano et al., 1997). However, the *set1A* and *set1B* genes overlap with the *pic* gene but are divergently transcribed. Therefore, the additional *pic*^−^ knockout may have caused these effects. Faherty et al. (2012b) complemented the *pic/set1AB* mutant with *pic* and *set1AB* individually, showing that *pic* has a more significant contribution to restoring I_sc_ levels to wild-type, although *set1AB* complementation also produced a significant increase in I_sc_.

ShET2 is a 63 kDa protein encoded by *ospD3* (*sen*). It is found in all serotypes and one of the three *ospD* genes found on the virulence plasmid. Sequence alignments between *ospD2* and *ospD3* show a high degree homology while *ospD1* is more divergent. OspD1 has a unique role in regulating type III secretion not shared with OspD2 and OspD3 (Parsot et al., 2005), but redundancy in their effector function(s) is unknown. Unlike ShET1, ShET2 secretion is dependent on the T3SS (Farfán et al., 2011) but how this is regulated unclear (Faherty et al., 2016). The *ospD3*^−^ (ShET2) knockout has similar effects in Using chamber experiments to a *set1AB*^−^ (ShET1) knockout, with reduced I_sc_ increase in comparison to the wild-type strain (Nataro et al., 1995). An *ospD3*^−^ mutant also had a reduction in IL-8 secretion, which could be restored to wild-type levels by *ospD3* plasmid complementation, indicating a possible role in IL-8 secretion by epithelial cells (Farfán et al., 2011).

### Adhesion to the colonic epithelium at the basolateral surface

#### Lipopolysaccharide: glucosylation for T3SS accessibility

The lipopolysaccharide (LPS) is a common feature of Gram negative pathogens, triggering the host immune response and inflammatory reactions during infection. LPS modification by glucosylation is thought to contribute to *Shigella* adhesion and invasion by revealing the T3SS for efficient activation upon contact with the host cell. Guan et al. (1999) showed that glycosyltransferase *gtrA*^−^ and *gtrB*^−^ mutants had only a partial conversion of the O-antigen serotype, and a *gtrX*^−^ mutant had no conversion at all. A mutation in the *gtr* operon leads to a reduced ability to invade, and this invasion is restored when the *gtr* operon is reintroduced (West et al., 2005). The reduction in O-antigen length by glucosylation enhances accessibility of the T3SS for contact with the host epithelial cell to initiate invasion.

#### IpaB: binds CD44 at the basolateral surface

IpaB mediates adhesion to the basolateral membrane via interactions with the ubiquitous glycoprotein CD44 (Figure 1, step 7). CD44 is located within lipid microdomain rafts, and is involved in binding of ezrin, radixin, and moesin (ERM) proteins to produce rearrangements of the actin cytoskeleton. IpaB binds the CD44 N-terminal domain with weak affinity but up-regulation of CD44 expression to levels found on lipid microdomains increases binding and internalization of *Shigella* (Skoudy et al., 2000). Lipid microdomain rafts are found at the basolateral surface and the IpaB-CD44 adhesion interaction may contribute to the polarity of *Shigella* invasion of epithelial cells (Lafont et al., 2002). Although increased adherence mediated by IpaB-CD44 binding may improve invasion efficiency, this binding alone is not sufficient to induce *Shigella* entry, as both *ipaC*^−^ and *ipaD*^−^ (see below) mutants are unable to mediate invasion (Skoudy et al., 2000).

#### IcsA (VirG): polar adhesion

**Figure 4:**
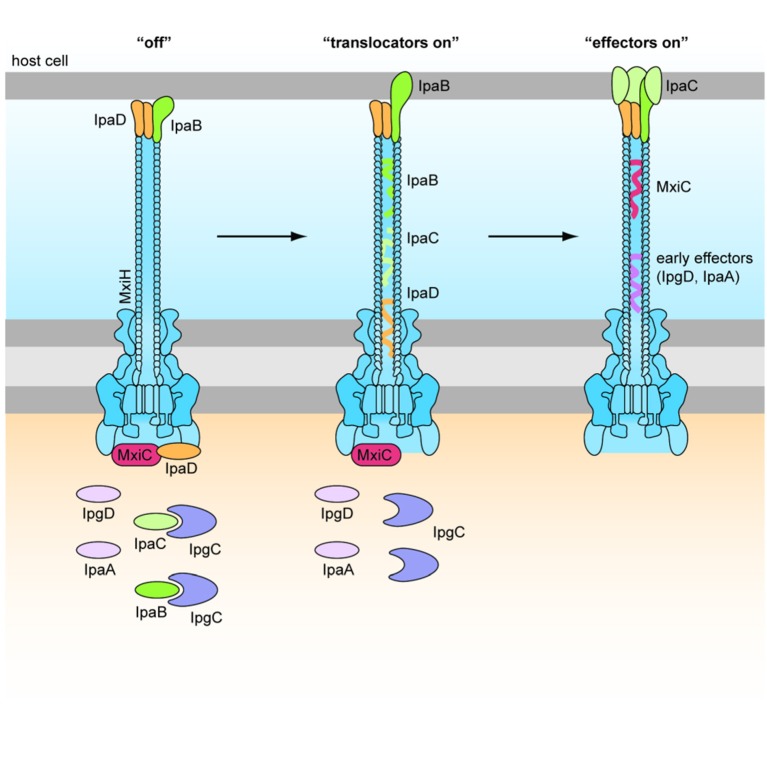
**Role of IpaD, IpaB, and IpaC in regulation of the T3SS (Martinez-Argudo and Blocker, 2010)**. Four hydrophilic IpaD molecules and one hydrophobic IpaB molecule are localized at the tip of the T3SS needle in the “off” state. IpaB senses host cells upon contact of the T3SS needle tip, and inserts into the membrane to signal “translocators on.” Secretion of effectors is signaled by conformational changes in IpaD via a signal transduction pathway to the base of the secreton. Four IpaC may travel up the secreton and associate with the needle tip, one atop each IpaD, and insert around the single IpaB to form a translocon in the epithelial cell membrane. This signals “effectors on” and early effectors are injected into the cytoplasm of the host epithelial cell (Veenendaal et al., 2007).

**Figure 5:**
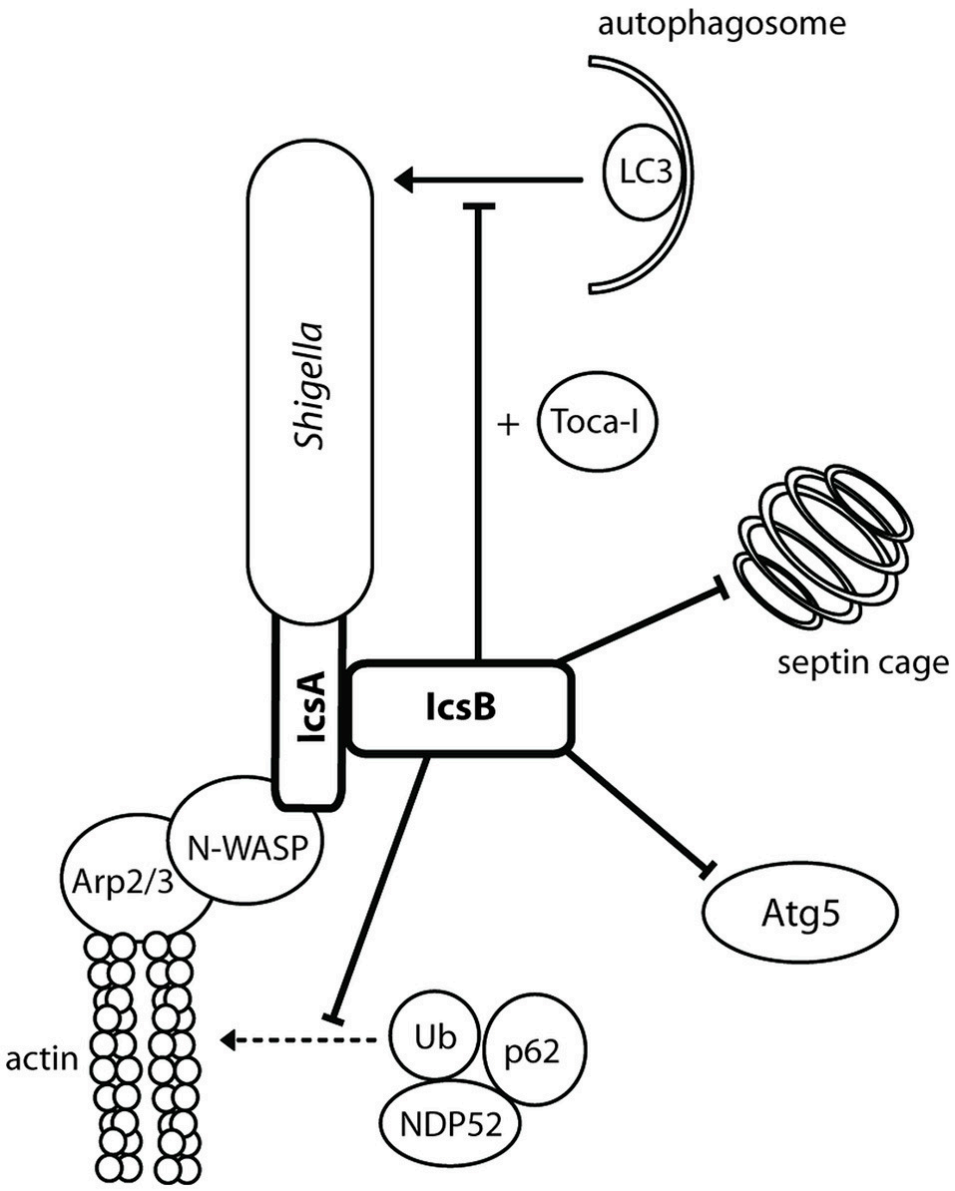
**IcsA mediates actin-based motility and IcsB prevents autophagy**. IcsA is localized in a unipolar fashion to promote efficient unidirectional intracellular movement parallel to the long axis of *Shigella* for movement intra- and intercellularly. IcsB masks the Atg5 recognition site on IcsA, recruits Toca-I, and prevents the formation of septin cages.

IcsA, also referred to as VirG, is a 120 kDa outer membrane protein. IcsA is not dependent on the T3SS for its secretion as it is an autotransporter, with an atypical N-terminal signal sequence mediating secretion via the Sec pathway (Brandon et al., 2003). It is most well-known for its involvement actin based motility, however a more recent function has been described, whereby IcsA is involved in polar adhesion of *Shigella* to epithelial cells (Brotcke Zumsteg et al., 2014). The adhesion function can be separated from actin-based motility, as an *icsA*^−^ mutant complemented with a plasmid encoding an adhesion-defective *icsA* formed plaques similar to wild-type *Shigella* (Brotcke Zumsteg et al., 2014). The adhesion-defective *icsA* also produced an attenuated infection phenotype in the Sereny test, indicating the importance of IcsA as an adhesin in *Shigella* pathogenesis. IcsA-mediated adhesion was present in an *ipaBCDA*^−^*mxiE*^−^ mutant, but not in an *ipaD*^−^*spa33*^−^ strain, indicating that the assembled T3SS, but not the secretion of T3SS effectors, is required for adhesion activity. This initial observation was then linked to T3SS-dependent activation of IcsA to mediate this adhesive phenotype, as the application of the bile salt deoxycholate (DOC) led to an increase in IcsA-dependent adhesion (Brotcke Zumsteg et al., 2014). Deoxycholate has previously been described to bind to IpaD at the T3SS needle tip and induce IpaB recruitment for T3SS activation (Barta et al., 2012). However, DOC is also known to effect LPS molecules, causing them to disperse within the membrane (Shands and Chun, 1980). Assessment of protease accessibility using proteinase K with an *ipaD*^−^ mutant and after DOC treatment showed that hyperadhesive IcsA was more resistant to degradation, and this was proposed to be due to an alternate conformation of IcsA induced by T3SS activation (Figures 4C, 5D—Brotcke Zumsteg et al., 2014). However, as DOC does not activate the T3SS, the changes in proteolysis banding are more likely due to disruption of the LPS leading to altered accessibility of IcsA producing different cleavage patterns. Changes seen in the *ipaD*^−^ mutant could occur via an independent regulatory pathway, whereby activation of the T3SS leads to modulation of LPS structure.

#### OpsE1/E2: bile salts-dependent adhesion

Faherty et al. (2012a) also noticed that subculture in media containing bile salts significantly enhanced ability of *Shigella* to adhere to the apical surface of polarized epithelial cells. However, microarray expression analysis indicated that the *ospE1*/*ospE2* genes were induced in the presence of bile, and bile-induced adherence was lost in a Δ*ospE1*/Δ*ospE2* mutant. The OspE1/OspE2 proteins, which are effectors secreted by the T3SS, were also shown to remain localized to the bacterial outer membrane following exposure to bile salts, where they may therefore serve as adhesins.

### Macropinocytic uptake into colonic epithelial cell

#### IpaD and IpaB: activation of the T3SS

IpaD is part of the Ipa family required for *Shigella* invasion, and polymerizes at the T3SS needle tip (Espina et al., 2006). The identification of Class I *ipaD*^−^ mutants, which had premature secretion of effectors, and Class II *ipaD*^−^ mutants, which were non-inducible, shows that IpaD has a dual role in the activation of the T3SS (Roehrich-Doenitz et al., 2013). IpaD acts as the scaffold protein at the tip of the T3SS needle acting as the display support for IpaB, located at the needle tip along with IpaD (Veenendaal et al., 2007), and the delivery mechanism of the hydrophobic IpaB-IpaC translocation pore (translocon) to host cell membranes (Blocker et al., 1999). From there IpaD acts as a signal transducer to activate effector secretion (Figure 4) (Roehrich-Doenitz et al., 2013). IpaD, in conjunction with MxiC, is also part of the cytoplasmic signal transduction pathway required for full activation of the T3SS and secretion of remaining T3SS effectors (Martinez-Argudo and Blocker, 2010). IpaB initially senses the host cell membrane in a manner that is not yet understood and, with IpaD, co-transduces this signal down the T3SS needle to activate secretion (Murillo et al., 2016).

#### IpaC: actin polymerization and induction of effector translocation

IpaC belongs to the group of Ipa proteins crucial for translocon formation and cell invasion. The structure of IpaC includes an N-terminal signal sequence, a region for association with IpgC (cytoplasmic chaperone), a central hydrophobic region for penetration of membranes, and a C-terminal domain for oligomerization (Terry et al., 2008). The hydrophobic region allows IpaC to interact with IpaB and insert into host membranes, however its topology in the membrane is disputed. The use of anti-IpaC monoclonal antibodies has shown that both the N- and C-terminal regions face the host cell cytoplasm (Tran Van Nhieu et al., 1999) although other experiments have found that the central loop is on the cytoplasmic face, with the N- and C-terminal regions being extracellular (Kuwae et al., 2001). IpaC insertion into the epithelial cell membrane triggers cytoskeletal rearrangements for the macropinocytic uptake of *Shigella* (Figure 4). Menard et al. (1996) describe how an IpaB-IpaC complex on the surface of latex beads is sufficient for engulfment by non-phagocytic cells. However, this has not been reproduced and therefore may not applicable *in vivo*. It is likely that the IpaC C-terminus faces the host cytoplasm because IpaC has been attributed the ability to polymerize actin indirectly at its C-terminus, via interactions with Cdc42 and Rac GTPases (Tran Van Nhieu et al., 1999) and activation of Src tyrosine kinase pathway (Mounier et al., 2009). However, the *Salmonella* homolog, SipC, can polymerize actin at its C-terminal domain, independently of any host cell factor (Hayward and Koronakis, 1999). IpaC displays sequence similarity to SipC within its C-terminal actin nucleation domain, and both IpaC and SipC use this domain to oligomerize. IpaC may therefore also be capable of polymerizing actin independently. In pull down assays, the C-terminal domain of IpaC was incapable of binding to Cdc42 and Rac1, indicating that actin polymerization does not occur through direct interactions with these GTPases (Terry et al., 2008). Finally, the C-terminus of IpaC has also been shown to bind vimentin and the intestinal epithelial intermediate filament keratin 18. This interaction is required for stable docking of the bacteria to cells and a prerequisite for induction of secretion of the other effectors (Russo et al., 2016).

#### IpgB1 and IpgB2: actin remodeling

IpgB1 and IpgB2 share 25% amino acid identity and both require Spa15 as a chaperone for secretion, with an additional requirement for stability by IpgB1 (Hachani et al., 2007). They contain a WxxxE motif which is common in guanine nucleotide exchange factors (GEFs) involved in the activation of Rho GTPases. These GTPases are required for the induction of actin filament structures to produce membrane ruffles for bacterial entry into non-phagocytic cells. An *ipgB1*^−^ knockout has a 50% reduction in epithelial cell invasion compared to the wild type stain, which was restored when complemented by plasmid-expression of *ipgB1* (Ohya et al., 2005). Membrane ruffle size is also affected, as wild-type strains achieved ruffle size of 60 μm^2^, an *ipgB1*^−^ mutant achieved 16 μm^2^, and an *ipgB1*-hyperproducing strain achieved 138 μm^2^ (Ohya et al., 2005). This indicates that IpgB1 is involved in the production of membrane ruffles in a dose-dependent manner. It was disputed as to whether IpgB1 activated Rac1 directly (Alto et al., 2006) or whether IpgB1 mimicked RhoG for activation of Rac1 indirectly via the ELMO-Dock180 pathway (Handa et al., 2007). However, crystal structures of IpgB1 confirmed that it acts as a GEF, specifically recognizing the β2-3 residues of Cdc42 and Rac1 GTPases to catalyse the GDP-GTP exchange for activation (Huang et al., 2009). Crystal structures and functional studies also confirmed that IpgB2 is a GEF capable of directly binding and activating RhoA (Huang et al., 2009; Klink et al., 2010). Activation of Rac1 and RhoA by IpgB1 and IpgB2 contributes to formation of lamellipodia and actin stress fibers, respectively. In the Sereny test, an *ipgB2*^−^ mutant produced the same disease phenotype as the wild-type strain, and a negative result only occurred in an *ipgB1*^−^*ipgB2*^−^ mutant, indicating redundancy (Hachani et al., 2007). However, an *ipgB1*^−^ mutant alone produced a more severe inflammatory phenotype than the wild-type strain, which was unexpected.

#### IpaA: actin depolymerization

IpaA is involved in regulating actin protrusions at the epithelial membrane and depolymerization of actin filaments in the host cell during *Shigella* entry. This is postulated to be achieved through its interactions with vinculin, a host protein that links the cytoskeleton to the extracellular matrix and is involved in focal adhesion structures. There are three vinculin binding sites, which are arranged tandemly at the IpaA C-terminus, each of which can bind one vinculin head. Binding of IpaA to the vinculin head induces a conformational change in vinculin, revealing an F-actin binding site in the vinculin tail (Izard et al., 2006). IpaA is important for cell entry, as an *ipaA*^−^ mutant has a 10-fold decrease in invasion capacity, and requires vinculin to mediate its effects, as vinculin-deficient cells had a similar invasion defect (Tran Van Nhieu et al., 1997). Pelleting assays showed that no actin depolymerization occurred in the presence of IpaA or vinculin alone, with actin principally in the pellet, but when the IpaA:vinculin complex was added, actin was found mostly in the supernatant, with the amount of depolymerization correlating with vinculin concentration (Bourdet-Sicard et al., 1999). The IpaA:vinculin complex has a 3-fold increased affinity for F-actin compared to vinculin alone and acts as a “leaky cap” on the barbed end of the F-actin filaments to prevent addition of further monomers and cause depolymerization (Bourdet-Sicard et al., 1999; Ramarao et al., 2007). IpaA therefore prevents the uncontrolled formation of IpaC-induced microspike structures at the site of bacterial contact, which would repel *Shigella* from the epithelial cell surface (Tran Van Nhieu et al., 1997).

#### IpgD: membrane ruffles

IpgD plays an important role in the formation of bacterial entry structures on contact with host epithelial cells. An *ipgD*^−^ mutant induces a less efficient entry structure than the wild type, due to smaller membrane ruffles and a reduction in actin rearrangements (Niebuhr et al., 2000). IpgD functions as an inositol (phosphoinositide) 4-phosphatase, and has sequence motifs similar to mammalian phosphoinositide phosphatases and *Salmonella* homolog SopB/SigD with similar inositol phosphate phosphatase activity (Niebuhr et al., 2002). Its main substrate in the host cell is phosphatidylinositol 4,5-bisphosphate [PtdIns(4,5)P_2_/PIP2] which it desphosphorylates to produce phosphatidylinositol 5-monophosphate [PtdIns(5)P/PI5P]. An *ipgD*^−^ mutant, or IpgD with a cysteine to serine (C438S) substitution in its active site, has no affect on PtdIns(4,5)P_2_ levels (Niebuhr et al., 2002). The dephosphorylation activity of IpgD on PtdIns(4,5)P_2_ correlates with a decrease in membrane tether force, as PtdIns(4,5)P_2_ controls the adhesion force between the plasma membrane of the epithelial cell and the actin cytoskeleton (Niebuhr et al., 2002). Combined with actin cytoskeleton rearrangements mediated by other *Shigella* effectors, the reduction in membrane tether allows the extension of filopodia and membrane ruffles characteristic of the trigger mechanism seen in *Shigella* entry into non-phagocytic cells (Figure 1, step 7) (Niebuhr et al., 2002). IpgD inositol-4 phosphatase activity has also been implicated in a positive feedback loop involving ARF6 GTPase (Garza-Mayers et al., 2015), which stimulates actin remodeling and membrane ruffles through Rac1 activation. The production of PtdIns(5)P by IpgD activates phosphoinositide 3-kinase (PI3K), which generates PtdIns(3,4,5)P_3_. This recruits ARF nucleotide binding site opener (ARNO), a GEF that activates ARF6 GTPase. Active ARF6-GTP promotes actin remodeling through Rac1-dependent pathways, further contributing to membrane ruffles for *Shigella* entry.

### Replication and spread within colonic epithelium

#### IpaB and IpaC: lysis of single membrane entry vacuole?

Due to the pre-requisite for invasion of epithelial cells and time coupling between entry and vacuolar lysis (15 min), disruption of components required for entry can seem to have pleotrophic effects on downstream infection events. This creates intrinsic limitations in studying their role in intracellular pathogenesis (Guichon et al., 2001). Nonetheless, an “entry region” encoding only the T3SS and IpaD, IpaB, and IpaC has been demonstrated to be sufficient for vacuolar lysis (Figure 1, step 8) (Sansonetti et al., 1986; Du et al., 2016). High et al. (1992) used macrophages, which are naturally phagocytic, to overcome the non-invasive *ipaB*^−^ phenotype, and described how IpaB plays a role in lysis of the phagocytic vacuole. It has been postulated that the insertion of the IpaB-IpaC translocon into the vacuolar membrane is the cause of membrane lysis (High et al., 1992). This would occur through pore formation, which could lead to vacuolar destabilization, or through translocation of unidentified membranolytic effector(s) across the membrane (Page et al., 1999). Senerovic et al. (2012) found that purified IpaB internalized into cells oligomerized in endocytic membranes to form ion channels which affected their integrity. However, the IpaB used was purified recombinantly using a detergent, which naturally lyses membranes, and this effect was not controlled for. Moreover, when within the naturally inserted translocon IpaB is connected to the T3SS via the needle tip, which - along with low osmolarity in the vacuole - would prevent the influx of water into the vacuole to cause lysis. Furthermore, by exploring the functional interchangeability of translocon components from *Shigella* and *Salmonella*, which remains in its invasion vacuole, IpaC was shown to be directly involved in lysis of the single membrane vacuole (Osiecki et al., 2001; Du et al., 2016). Yet, any environmental cue for the translocon to switch between possible invasion, translocation, and lysis conformations remains unknown.

#### IpgD: lysis of single membrane entry vacuole?

**Figure 6:**
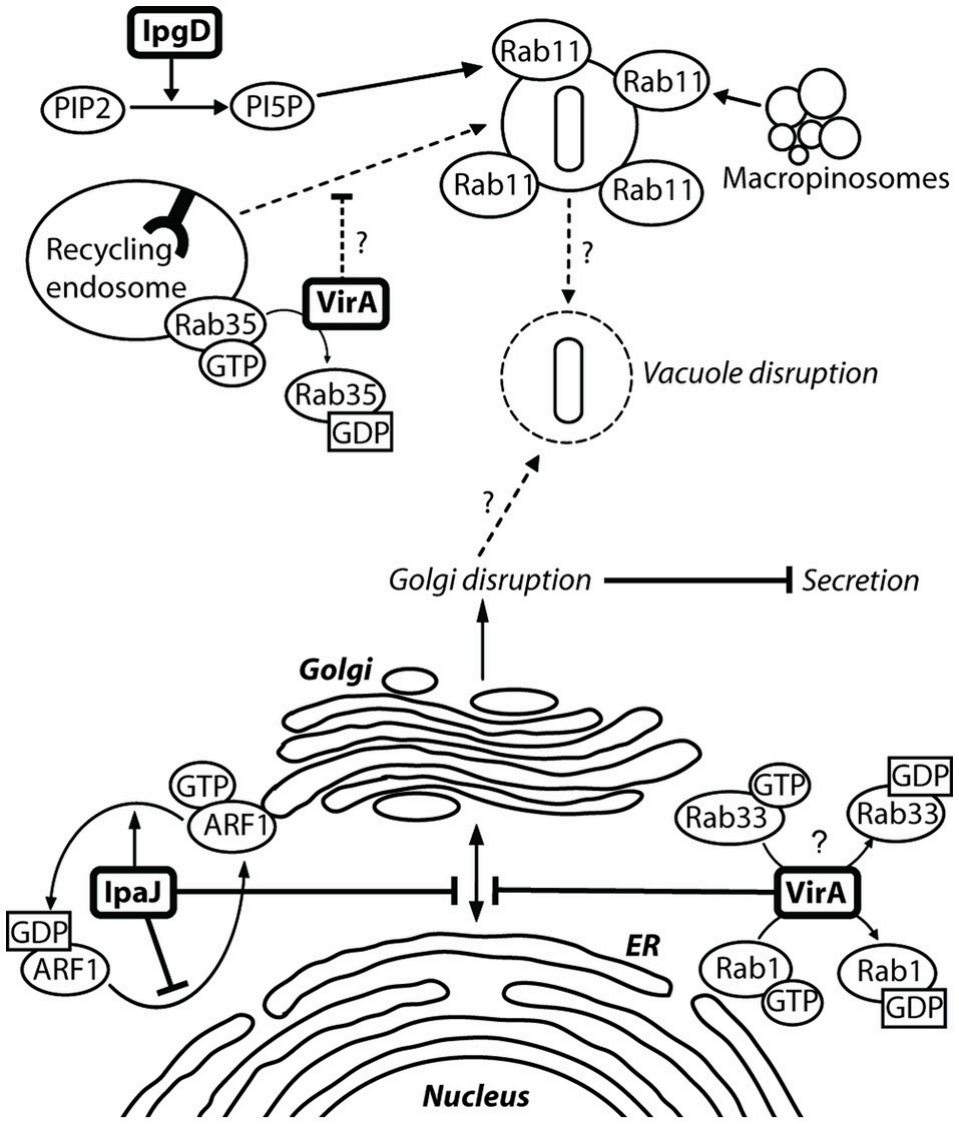
**VirA and IpaJ mediate Golgi fragmentation, which may contribute to entry vacuole disruption**. There is a level of semi-redundancy between VirA and IpaJ, as a double *virA*^−^*ipaJ*^−^ knockout is required to completely abolish *Shigella*-induced Golgi fragmentation. IpaJ is the dominant effector, and targets ARF1-GTP for N-myristoylated cleavage at the exposed di-glycine motif. ARF1 is irreversibly lost from the Golgi membrane, leading to inhibition of Golgi trafficking (Burnaevskiy et al., 2013). VirA is capable of interacting and inactivating many Rab GTPases to mediate Golgi fragmentation, including Rab1 at the ER and Rab33 at the Golgi (Dong et al., 2012). This causes disruption of ER-to-Golgi trafficking, leading to fragmentation of the Golgi. Inactivation of Rab35 at the recycling endosome may prevent recruitment of additional membrane to the *Shigella* entry vacuole. Replication of *Shigella* within the entry vacuole without additional membrane may then allow vacuole lysis. IpgD may also contribute to vacuole lysis by recruiting Rab11 to macropinosomes, which have been shown make contact with the entry vacuole to accelerate vacuolar rupture (Mellouk et al., 2014; Weiner et al., 2016).

IpgD may be involved in the modulation of the *Shigella*-induced entry vacuole by recruiting Rab11 to macropinosomes. An siRNA screen and Rab11-depleted cells showed that the absence of Rab11, a small GTPase involved in endocytic recycling, leads to decelerated *Shigella*-induced vacuolar rupture. Like the Rab11-depleted cells, vacuolar rupture was delayed in an *ipgD*^−^ mutant, taking twice as long as for the wild-type strain (Mellouk et al., 2014). Functionally impaired Rab11 and a GDP-locked dominant negative Rab11 showed that it is the absence of Rab11 activity that causes this delay in vacuolar rupture (Weiner et al., 2016). Immunofluorescence staining co-localized Rab11-positive vesicles at the *Shigella* invasion site and *Shigella*-containing vacuoles, however this accumulation did not occur if an *ipgD*^−^ mutant or IpgD lacking its inositol-4 phosphatase activity was present in the vacuole (Mellouk et al., 2014). Using C-FIB/SET, Weiner et al. (2016) then showed that the Rab11-positive vesicles are macropinosomes, which are formed during membrane ruffling induced by *Shigella*. IpgD is involved in macropinosome formation, through its stimulation of ruffling, and hence in making these organelles available to the *Shigella* entry vacuole (Weiner et al., 2016). Macropinosomes are required for efficient entry vacuole rupture and have been visualized close to the entry vacuole, making direct contact just prior to vacuolar rupture (Weiner et al., 2016). This suggests that the phosphoinositide phosphatase activity of IpgD is required to regulate Rab11 recruitment to macropinosomes for attachment to the *Shigella*-containing entry vacuole (**Figure 6**). How macropinosome attachment, which does not lead to fusion with the vacuole, leads to rapid vacuolar rupture is unknown.

#### IcsA (VirG): actin-based motility

IcsA is an autotransporter and is composed of three domains: An N-terminal signal sequence, a C-terminal β barrel core which forms a pore in the outer membrane, and a central α-domain which is translocated through the β core membrane pore and present at the *Shigella* surface (Suzuki et al., 1995). Surface exposed IcsA is sometimes cleaved, however this is not required for IcsA function (Fukuda et al., 1995). The importance of IcsA in *Shigella* intercellular spread was identified early, as an *icsA*^−^ mutant was unable to spread within an epithelial monolayer (as measured by plaque formation) and had a negative Sereny test (Bernardini et al., 1989). IcsA-mediated actin based motility is sufficient for membrane protrusion formation (Figure 1, step 10) and entry into neighboring cells (Figure 5). IcsA acts as a mimic of Cdc42 to activate N-WASP, which allows the N-WASP C-terminus to recruit Arp2/3 (Egile et al., 1999; Shibata et al., 2002). This promotes rapid F-actin assembly and filament growth at the N-terminus of N-WASP, providing a propulsive force for *Shigella* to move through the cell. When *icsA* is expressed in *E. coli*, these bacteria are capable of forming membrane protrusions with similar morphology to *Shigella*-induced protrusions, indicating that no other *Shigella* factors are required for this process (Monack and Theriot, 2001). *Shigella* factors, such as IcsP (SopA), are required for correct localization and cleavage of IcsA at the *Shigella* surface, contributing to efficient motility (Egile et al., 1999). On addition of *icsP* to *icsA*-expressing *E. coli* there was an increase in actin polymerization and increase protrusion frequency. Furthermore, in *E. coli* LPS O-antigen mutants there was a decrease in formation of actin tails compared to the wild-type. A *galU*^−^ mutant, which normally encodes a UDP-glucose pyrophosphorylase involved in O-antigen biosynthesis, produces a diffuse circumferential pattern of IcsA on the *Shigella* surface, which is still capable of polymerizing actin but forms no membrane protrusions (Sandlin et al., 1995). However, it is unknown how the LPS modulates IcsA localization. Therefore, both IcsP and LPS are required for the unipolar localization of IcsA to produce efficient unidirectional movement, which is strongly correlated with frequency of membrane protrusions (Monack and Theriot, 2001).

#### IcsB: inhibition of autophagy

IcsB requires the IpgA chaperone for both its stability and its secretion (Ogawa et al., 2003). An *icsB*^−^ mutant produced plaques with a smaller diameter than the wild type and a negative result in the Sereny test, suggesting a role for IcsB post-invasion. This role is the prevention of autophagy (Figure 5). IcsB prevents autophagic recognition by masking the Atg5 binding site on IcsA, preventing Atg5 from binding and initiating autophagosome formation. IcsB is also capable of recruiting Toca-I to prevent LC3-mediated phagocytosis (Baxt and Goldberg, 2014). IcsB prevents the formation of septin cages, which in turn may prevent the recruitment of ubiquitin (Ub), p62 and NDP52 (Huang and Brumell, 2014). In an *icsB*^−^ knockout, autophagic double membranes were visualized around the *Shigella* bacilli, with asymmetric distribution similar to IcsA placement (Ogawa et al., 2005). IcsB, in conjunction with VirA, has also been implicated in lysis of the double membrane entry vacuole after intercellular spread (Figure 1, step 11). Electron microscopy (EM) 3 h after cell infection visualized *icsB*^−^ mutants remaining trapped in a double membrane, with several bacteria in one vacuole (Allaoui et al., 1992). Galectin-3 had then been used to show that an *icsB*^−^ mutant has only a 53% disruption of the double membrane vacuole, compared to 70% disruption mediated by the wild type (Campbell-Valois et al., 2015). However, on closer inspection, we think the EM images (Allaoui et al., 1992, Figure 8B) were misinterpreted, and the double membrane interpreted as the secondary entry vacuole was actually a starting autophagosome wrapping around the *icsB*^−^ mutant unable to inhibit autophagy. Furthermore, galectin-3 can be used to label endomembranes or autophagic membranes, as it interacts with β-galactose-containing glycoconjugates which are present in both endocytic and secretory compartments (Maejima et al., 2013). Therefore, instead of rupturing the secondary entry vacuole IcsB inhibits autophagy of *Shigella*.

#### VirA: inhibition of autophagy and promotion of golgi fragmentation

VirA was initially thought to play a role in *Shigella* invasion, as a *virA*^−^ mutant had a 5-fold reduced capacity for invasion (Uchiya et al., 1995). This was linked to its apparent cysteine protease activity and capability for microtubule degradation (Yoshida et al., 2002). However, structural analysis showed that VirA lacks the suggested papain-like protease activity for tubulin cleavage, and instead exhibits homology with EspG, an EPEC effector that fragments the Golgi (Germane et al., 2008). VirA belongs to a family of GTPase-Activating Proteins, which share the conserved Rab GTPase catalytic Tre-2/Bub2/Cdc16 domain to mediate Rab1 GTP hydrolysis (Dong et al., 2012). Rab1 GTPase is involved in ER-to-Golgi vesicular transport and is crucial in the formation of autophagosomes (Zoppino et al., 2010). VirA stabilizes Rab1 in the inactive GDP state, thereby directly interfering with autophagy induction and ER-to-Golgi trafficking (Figure 6) (Dong et al., 2012; Huang and Brumell, 2014). A *virA*^−^ mutant leads to reduced *Shigella* intercellular persistence, but does not greatly reduce Golgi fragmentation, as IpaJ is more potent in fragmenting the Golgi (Figure 6) (Dong et al., 2012; Burnaevskiy et al., 2013). VirA, similarly to IcsB, has been implicated in the disruption of the secondary vacuole after intercellular spread following membrane protrusion formation (Figure 1, step 10) (Campbell-Valois et al., 2015). However, like for IcsB, this may have been misinterpreted. In our view, the evidence indicates that VirA is involved in lysis of the single membrane entry vacuole (Figure 1, step 8). Indeed, Lysosomal Associated Membrane Protein 2 (LAMP2), a marker for lysosomal fusion with the entry vacuole, has been localized by confocal microscopy to entry vacuoles containing a single *virA*^−^ mutant and a double *icsB*^−^*virA*^−^ mutant (Campbell-Valois et al., 2015, Figures 2B, 3C) (Dong et al., 2012, Figure S3). A single *icsB*^−^ mutant is capable of escaping the entry vacuole, visualized by actin comet tail formation (Allaoui et al., 1992, Figure 8A). Therefore, the lack of escape from a single membrane entry vacuole by the double *icsB*^−^*virA*^−^ mutant (Campbell-Valois et al., 2015, Figure 4) can be attributed to loss of VirA function. To confirm this, EM analysis of a single *virA*^−^ mutant is required. VirA may mediate vacuolar lysis through an indirect mechanism, whereby its inhibition of endosomal trafficking prevents membrane vesicle fusion to the entry vacuole (Figure 6). This could occur through interactions with multiple Rab GTPases, as *in vitro* assays have indicated that VirA can bind many Rab proteins involved in ER-to-Golgi traffic and recycling (Dong et al., 2012). The vacuole may consequently lyse as it cannot grow to accommodate *Shigella* replication. Passive lysis of the vacuole has been shown for *Salmonella*, whereby a *sifA*^−^ knockout prevents the recruitment of membrane to the entry vacuole. The vacuole cannot sustain *Salmonella* replication and subsequently lyses, releasing *Salmonella* into the epithelial cytoplasm (Beuzón et al., 2000).

#### IpaJ: golgi fragmentation

*ipaJ* is encoded downstream of the *ipaBCDA* operon and transcribed divergently to it. But the original *ipaJ*^−^ mutant showed no defect in plaque formation and was Sereny test positive. Therefore, it did not seem to play a crucial role in epithelial invasion or cell-to-cell spread (Buysse et al., 1997). Structural bioinformatics analysis indicated that IpaJ harbored catalytic residues required for peptide bond hydrolysis, and further experiments identified that IpaJ is a cysteine protease which preferentially cleaves N-myristoylated proteins (Burnaevskiy et al., 2013). Although *in vitro* studies indicated that IpaJ has a large spectrum of N-myristoylated targets (Burnaevskiy et al., 2015, Figure 1D), *in vivo* it specifically targets ADP-ribosylation factors (ARF), particularly ARF1 (Burnaevskiy et al., 2015, Figure 4D). ARF1 GTPase is localized to the Golgi membrane and the plasma membrane as it plays a role in ER-to Golgi transport, including vesicle formation for cargo transport and maintenance of the Golgi (D'Souza-Schorey and Chavrier, 2006). Removal of the myristoyl group from GTP-active ARF1 by IpaJ causes its irreversible release from the Golgi, inhibiting vesicular trafficking (Figure 6). An *ipaJ*^−^ mutant has no effect on the ARF1 intracellular pool, but the wild-type strain decreases the amount of ARF1 GTPase bound to the Golgi (Burnaevskiy et al., 2015). Mounier et al. (2012) suggested that IpaB mediates Golgi fragmentation via modulation of the membrane cholesterol concentration, and state that VirA has no obvious effect on disruption. However, the effects of IpaJ were not accounted for, and as the dominant effector in this semi-redundant pair, it is likely that effects attributed to IpaB were actually mediated by IpaJ. The consequences of Golgi disruption by IpaJ are not fully understood but inhibition of STING relocalization from the endoplasmic reticulum (ER) to the intermediate compartment between ER and Golgi (ERGIC) may be one them (Dobbs et al., 2015). STING is a major sensor of cytoplasmic pathogens through detection of DNA and cyclic dinucleotides, where upon it translocates from the ER to ERGIC and activates of the IFN-I pathway.

#### OspE1 and OspE2: promotion of host cell adherence to basement membrane

OspE1 and OspE2 are 99% identical, which suggests they may have arisen from a gene duplication event (Buchrieser et al., 2000). They are capable of functioning redundantly, however in *S. sonnei, ospE1* is a pseudogene (Miura et al., 2006). An *ospE2*^−^ knockout caused cellular rounding that was not as a result of apoptosis or necrosis, and wild-type phenotype was restored when *ospE2* knockouts were complemented with functioning *ospE2* encoded on a plasmid (Miura et al., 2006). OspEs are capable of interacting with integrin-linked kinase (ILK), which is found in the membrane of host cells where it reinforces focal adhesions (Kim et al., 2009). The interaction between OspE and ILK reinforces adhesion contacts between the epithelial cell and the basement membrane (Miura et al., 2006). The OspE-ILK complex interferes with focal adhesion disassembly, reducing focal adhesion kinase phosphorylation and increasing surface β1 integrins (Kim et al., 2009). Tagged OspE was visualized at focal adhesions, however it was diffuse in ILK^−/−^ cells, indicating that ILK is required for OspE membrane localization, and this in turn increases the amount of ILK in the host membrane (Miura et al., 2006). An *in vivo* inoculation model in the distal colon of guinea pigs showed no *Shigella*-induced symptoms when a dual *ospE* knockout was used, however dual complementation of *ospE* restored the wild-type phenotype, with both inflammation and hemorrhaging (Kim et al., 2009).

#### IpaB: cell cycle arrest

IpaB has been linked to cell cycle arrest through interactions with Mad2L2, an anaphase promoting complex inhibitor (Iwai et al., 2007). Mad2L2 is involved in promoting entry of epithelial cells into mitosis during G2/M phase by interacting with the Cdh1, an anaphase promoting complex (APC) associated factor involved in preventing mitosis. After mitosis has occurred, Mad2L2 and Cdh1 dissociate and Cdh1 is activated to suppress mitotic cyclins. IpaB interferes with Mad2L2-Cdh1 binding, causing Cdh1 to be constitutively activated (Iwai et al., 2007). Permanent mitotic cyclin suppression by Cdh1 prevents epithelial turnover during *Shigella* infection, promoting more efficient bacterial replication by keeping the cells better attached to the adjacent cells and to the lamina. Cell cycle arrest thereby prevents epithelial cell turnover and allows *Shigella* to establish a better niche for replication. Interactions with Mad2L2 allow IpaB to be translocated into the epithelial nucleus (Iwai et al., 2007). IpaB binds Mad2L2, and this occurs at the same location on IpaB where the IpgC chaperone binds. Introduction of a single amino acid substitution conferring weaker binding of IpaB to Mad2L2 leads to a reduction in colonization of rabbit ileal loops suggesting that IpaB and its interaction with Mad2L2 contributes to more efficient *Shigella* colonization of the epithelium (Iwai et al., 2007, Figure 6B). However, the point mutation could instead have pleotropic effects on IpaB function (see IpaB and IpaC: Lysis of single membrane entry vacuole?) rather than directly affecting Mad2L2 binding.

### Lysis of the double membrane vacuole

#### Vps and VacJ: proposed ABC transporter

The *vpsABC* operon is found on the *Shigella* chromosome, and consists of VpsA, a possible ATP-Binding Cassette (ABC) transporter protein, and VspB and VspC, proposed transmembrane proteins. Both *vpsC*^−^ and *vspA*^−^ knockouts had a defect in plaque formation but were similar to wild-type strains in their capability to invade, indicating that they play a role in intercellular spread (Hong et al., 1998). VacJ is also encoded on the chromosome, and a *vacJ*^−^ knockout is incapable of escaping into the recipient cell cytoplasm, suggesting that VacJ also plays a role in intercellular spread (Suzuki et al., 1994). Carpenter et al. (2014) describe a Vps/VacJ ABC transporter, which maintains asymmetry of lipids in the outer membrane and in the context of *Shigella* infection is required for lysis of the double membrane vacuole. Transformation of *vps*/*vacJ* knockouts with a plasmid expressing *pldA*, a phospholipase in other Gram negative bacteria, was able to restore the maintenance of outer membrane lipid asymmetry but was unable to lyse the double membrane vacuole, indicating that these two functions of the proposed Vps/VacJ ABC transporter are separate (Carpenter et al., 2014). Another substrate may therefore be transported across the membrane to induce vacuolar lysis, however this is yet to be discovered.

#### IpaB and IpaC: translocon formation and lysis of the double membrane vacuole

Studying intracellular roles of IpaB and IpaC is difficult as their non-invasive mutants have pleotropic effects. However, Page et al. (1999) overcame this issue using a recombinant plasmid with IPTG-inducible *lac* promoter to regulate expression of IpaB and IpaC. After initial entry, IPTG was removed from the medium, producing *ipaB*^−^ and *ipaC*^−^ phenotypes which have a 3-fold decrease in plaque diameter compared to the wild-type. The inducible *ipaB*^−^ or *ipaC*^−^ mutants both exhibited a defect in lysis of the double membrane vacuole (Figure 1, step 11), with abolished membrane contact formation (Campbell-Valois et al., 2014). Several bacteria were visualized within such vacuoles, indicating that enough time was spent in the vacuole for replication to occur (Page et al., 1999). IpaB is located at the needle tip along with IpaD, from where it senses the host cell membrane (Murillo et al., 2016). Therefore, its interactions with the inner surface of the plasma membrane during membrane protrusion formation may induce the on-off regulation of the T3SS in the epithelial cell cytoplasm (Campbell-Valois et al., 2014) and fresh translocon insertion and/or translocation of unidentified effector(s) involved in lysis of the first or second membrane of the double membrane vacuole.

### Modulation of innate immune system

We cover here effectors directly involved in suppressing the innate immune response in epithelial cells and/or macrophages, rather than events up-stream of it discussed above, such as autophagy.

#### OspC3: inhibits caspase-4-mediated inflammatory cell death

**Figure 7:**
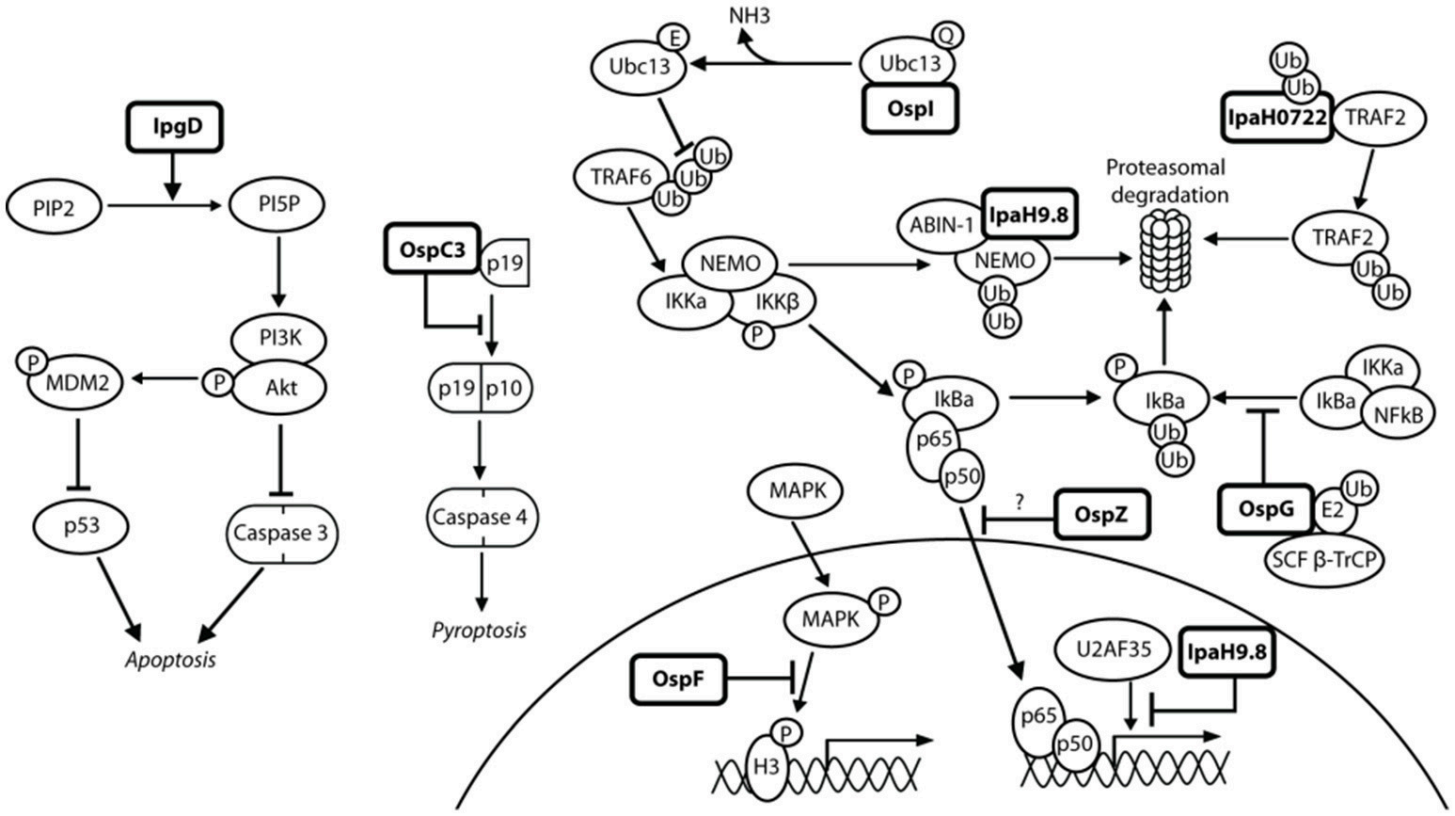
***Shigella* modulates the innate immune response in epithelial cells**. NFκB can be activated via many pathways during *Shigella* infection, including genotoxic stress during invasion and recognition of the lipopolysaccharide on the bacterial surface. This may explain the redundancy of *Shigella* effectors, many of which have the ability to interfere with these pathways, leading to efficient inhibition of NFκB. IpgD increases levels of PI5P, which activates PI3K and Akt and inhibits p53 and caspase 3. OspC3 sequesters the p19 pre-cursor of caspase 4, which prevents its heterodimerization with p10 and inhibits capase 4 activation. OspI deamidates Ubc13, which inhibits ubiquitination of TRAF6 and prevents activation of the TRAF6-NFκB pathway. IpaH0722 ubiquitylates TRAF2, leading to its proteasomal degradation and preventing recruitment of IKK and activation of NFκB. Polyubiquitination of NEMO/IKKγ is dependent on IpaH9.8 binding to A20 binding inhibitor of NFκB (ABIN-1), leading to proteasomal degradation of NEMO/IKKγ, activation of IκBα, and inhibition of NFκB. IpaH9.8 also localizes to the nucleus where it catalyses ubiquitination of U2AF^35^, leading to its degradation and consequently reduced *il-8* expression and decreased neutrophil recruitment. By an unknown mechanism, OspG prevents SCF^β−TrCP^ from ubiquitinating IκBα, preventing degradation of IκBα and activation of NFκB. OspZ blocks the nuclear translocation of p65, a subunit of NFκB, thereby inhibiting activation of NFκB. Threonine desphosphorylation of MAPKs by OspF leads to inactivation of ERK1/2 and p38 MAPK pathways and inhibition of histone H3 phosphorylation (H3pS10). This produces an inaccessible chromatin conformation at the gene promoters of inflammatory cytokines and chemokines, meaning NFκB cannot activate their transcription.

OspC3 is part of the *ospC* gene family. An *ospC3*^−^ mutant has an increased inflammatory cell death when compared to wild-type *Shigella* and *ospC1*^−^*/ospC2*^−^ mutants (Kobayashi et al., 2013). This indicates that OspC3 plays a role in the down-regulation of acute inflammatory cell death, and suggests a lack of redundancy in the *ospC* family as OspC1 has pro-inflammatory effects. Inflammatory cell death was not abolished with a cytochrome c or caspase-3/caspase-7 inhibitor. However, a caspase-1/caspase-4/caspase-5 inhibitor did reduce cytotoxicity and their activity increased during *ospC3*^−^ infection, suggesting that OspC3 mediates its activity via one of these caspase pathways (Kobayashi et al., 2013). Tagged OspC3 bound to the caspase-4 p19 subunit in a pull-down assay, and in-frame deletions showed that the terminal 190–484 residues of OspC3 were involved in p19 binding, specifically at a conserved consensus sequence, X_1_-Y-X_2_-D-X_3_ (Kobayashi et al., 2013). Substitution of all five residues with alanine, in addition to substitution of the conserved 450–478 residues in the C-terminal ankyrin region, impaired OspC3 binding to p19 and increased epithelial cytotoxicity (Kobayashi et al., 2013). Incubation of the p19 subunit with increasing concentrations of OspC3 correlated with increasingly impaired p19–p10 binding (Kobayashi et al., 2013). The biochemical function of OspC3 may therefore be interacting with p19 to inhibit caspase-4 activation and prevent inflammatory cell death (Figure 7).

#### OspF: inactivates MAPKs which prevents phosphorylation of histone H3

OspF was first described as a dual specific phosphatase (Arbibe et al., 2007), desphosphorylating threonine and tyrosine residues in the MAPK signaling pathway (Figure 7). However, it was determined by Li et al. (2007) through tandem mass spectrometry that OspF instead displays phosphothreonine lyase activity in that it irreversibly desphosphorylates threonine, but not tyrosine, through beta elimination (Zhu et al., 2007). An *ospF*^−^ mutant has increased PMN recruitment and severity of epithelial destruction in the rabbit ileal loop model when compared to the wild-type strain, indicating a role in down-regulating the immune response to *Shigella* infection (Arbibe et al., 2007). Phosphothreonine lyase activity is also seen in SpvC, a *Salmonella* homolog with 63% amino acid identity with OspF. Antibodies against phospho-amino acids confirmed specific removal of phosphate from threonine which inactivates MAPKs (Mazurkiewicz et al., 2008). OspF has also been attributed pro-inflammatory roles (Zurawski et al., 2006; Reiterer et al., 2011). The identification of accurate *in vivo* substrates may explain the apparent pro- and anti-inflammatory roles mediated by OspF.

#### OspG: inhibits NFκB activation

OspG plays a role in dampening the host immune response, shown by an *ospG*^−^ mutant exhibiting increased inflammation and destruction of the mucosa in comparison to wild-type *Shigella* in the rabbit ileal loop model (Kim et al., 2005). OspG has a minimal kinase domain, and its kinase activity requires binding of an E2 ubiquitin conjugating enzyme in conjunction with ubiquitin (Pruneda et al., 2014). Binding of UbcH7~Ub stabilizes OspG and confers an active kinase conformation, increasing kinase activity 20-fold (Grishin et al., 2014). OspG is also capable of binding UbcH5b~Ub, which is a component of the E3 ligating enzyme SCF^β−TrCP^ (Kim et al., 2005). An *ospG*^−^ mutant exhibits IκBα degradation 20 min post-invasion, whereas in wild-type *Shigella* this degradation occurs after 60 min (Kim et al., 2005). The exact mechanism of how OspG prevents SCF^β−TrCP^ from ubiquitinating phospho-IκBα is unknown, but the OspG kinase activity is postulated to be involved in the attenuation of NFκB activation (Figure 7) (Zhou et al., 2013).

#### OspI: inhibits NFκB activation

OspI functions as a glutamine deamidase, and has been shown to interfere with NFκB activation via the TNF-receptor-associated-factor (TRAF) 6 pathway (Sanada et al., 2012). An *ospI*^−^ mutant, when compared to wild-type *Shigella*, leads to increased levels of cytokine mRNA transcripts, increased phosphorylation of IκBα and a 4-fold increase in nuclear translocation of the p65 subunit of NFκB (Sanada et al., 2012). All of these lead to increased NFκB activation and consequently an increase in the host inflammatory response. OspI has a cysteine-histidine-aspartic acid catalytic triad which is crucial for deamidation, as activity was abolished by a cysteine-to-serine substitution (Sanada et al., 2012). A substrate of OspI is UBC13, an E2 ubiquitin conjugating enzyme required for TRAF6-induced NFκB activation, shown by binding of OspI to His-UBC13 during pull-down assays (Nishide et al., 2013). Hydrophobic interactions are important for this binding, and a crystal structure shows a glutamine residue at position 100 on UBC13 is positioned in the OspI active site (Nishide et al., 2013). OspI specifically deamidates Gln100, converting it to glutamic acid and abolishing the E2 ubiquitin conjugating function of UBC13 to prevent activation of the TRAF6-NFκB pathway (Figure 7) (Sanada et al., 2012).

#### OspZ: inhibits NFκB activation

As previously described, OspZ has a pro-inflammatory role in some *S. flexneri* species. In the remaining *Shigella* species the full length OspZ has an anti-inflammatory role, similar to that of its NleE homolog in EPEC (Newton et al., 2010). Both NleE and OspZ have been shown to block the nuclear translocation of p65, a subunit of NFκB, in response to TNFα and IL-1β (Figure 7). This leads to a reduction in transcription of pro-inflammatory cytokine genes, such as *il-8*, thereby reducing inflammation during *Shigella* infection. OspZ and NleE are also capable of inhibiting IκB degradation, further suppressing NFκB activity. Newton et al. (2010) determined that the crucial region for the anti-inflammatory effect of OspZ and NleE was between the amino acids 208–214, and has the sequence IDSYMK. Deletion of this region or single amino acid substitutions for alanine in NleE led to an increase in NFκB-dependent transcription (Newton et al., 2010, Figure 6D). NFκB activity was not abolished, indicating that this region is a binding site rather than an enzyme active site. The precise mechanism of how OspZ inhibits IκB degradation is unknown. However, Nadler et al. (2010) propose that NleE inhibits IKKβ, which is normally responsible for IκB phosphorylation and degradation in response to pro-inflammatory stimuli. OspZ may therefore work by a similar mechanism to prevent NFκB activation.

#### IpaH9.8: inhibits NFκB response

IpaH9.8 is a member of the *ipaH* gene family, and one of four found on the virulence plasmid, which includes *ipaH1.4, ipaH2.5, ipaH4.5*, and *ipaH9.8*. IpaH proteins are characterized by an N-terminal leucine-rich repeat (LRR) region and a highly conserved C-terminal region (CTR) which contains a cysteine residue (Suzuki et al., 2014a). The LRR motif is thought to play a role in protein-protein interactions, such as cell adhesion and signaling, while the cysteine residue in the CTR is required for enzyme 3 (E3) ubiquitin ligase activity. An *ipaH9.8*^−^ mutant has an increased inflammatory phenotype in comparison to wild-type *Shigella* in the murine lung model, indicating that IpaH9.8 has a role in attenuating inflammation during *Shigella* infection (Okuda et al., 2005). This is achieved through E3 ubiquitin ligase activity via the CTR of IpaH9.8 (Rohde et al., 2007). The substrates of IpaH9.8 include U2AF^35^and NEMO/IKKγ (Okuda et al., 2005; Ashida et al., 2010). Pull down assays confirmed IpaH9.8 and U2AF^35^ binding, which specifically occurs at the C-terminus of IpaH9.8 and 107–197 residues on U2AF^35^ (Okuda et al., 2005). Binding of IpaH9.8 to NEMO/IKKγ requires an ABIN-1 (A20 Binding Inhibitor) adaptor, as ABIN-1 knockdown leads to lack of IpaH9.8-induced effect on NEMO levels (Ashida et al., 2010). Ubiquitination of both U2AF^35^ and NEMO/IKKγ mediated by IpaH9.8 leads to their degradation in a proteasome-dependent manner (Figure 7) (Ashida et al., 2010; Perrett et al., 2011). Seyedarabi et al. (2010) describe how IpaH9.8 domain swapping occurs in response to host cell damage and this leads to dimerization and inactivation of its E3 ubiquitin ligase activity. *Shigella* may therefore sense the host cell conditions to maintain a suitable environment for its continued proliferation and survival.

#### IpaH0722: inhibits NFκB activation

IpaH0722 is encoded on the *Shigella* chromosome. In an *ipaH*-null mutant, whereby all seven of the chromosomal *ipaH* family genes were deleted, there was an increase in the severity of inflammation in the murine lung model in comparison to wild-type *Shigella* infection (Ashida et al., 2007). When individual *ipaH* knockouts were examined, it was found that IpaH0722 plays a role in dampening the inflammatory response, as an *ipaH0722*^−^ knockout had increased levels of IκBα degradation leading to NFκB activation (Ashida et al., 2013). IpaH0722 is also an E3 ubiquitin ligase, with the conserved CTR and crucial cysteine residue that is found in other *IpaH* proteins. A cysteine-to-alanine substitution increased NFκB activation, indicating that the E3 ubiquitin ligase activity is crucial for downregulation of NFκB (Ashida et al., 2013). Pull down assays showed that IpaH0722 could bind to TRAF2, however a CTR-truncation was unable to interact with TRAF2, further indicating the importance of IpaH0722 ubiquitin ligase activity (Ashida et al., 2013). IpaH0722 causes increased TRAF2 degradation leading to a reduction in NFκB activity, thereby dampening the host inflammatory response (Figure 7).

#### IpgD: activates Akt/PI3K signaling pathway

IpgD-mediated increase of PtdIns(5)P has been shown to induce Akt phosphorylation through activation of phosphatidylinositol 3-kinase (PI3K) (Mayo and Donner, 2001). An *ipgD*^−^ knockout has abolished Akt phosphorylation, which also occurs if PtdIns(5)P is sequestered or phosphorylated to PtdIns(4,5)P_2_ (Pendaries et al., 2006). Reduction in Akt phosphorylation was correlated with an increase in apoptosis and decreased phosphorylation of Mdm2, the negative regulator of p53, by the Akt serine-threonine kinase (Mayo and Donner, 2001). Bergounioux et al. (2012) showed that an *ipgD*^−^ mutant had a reduction in early phase Mdm2 phosphorylation, causing a delay in p53 degradation and increased apoptotic phenotype. The *Salmonella* homolog SopB and SigD also have pro-survival functions through interactions with Akt and activation of the PI3K-Akt survival pathway (Steele-Mortimer et al., 2000; Knodler et al., 2005).

### Macrophage vacuolar rupture and pyroptosis

#### IpaC: rupture of phagocytic vacuole

IpaC interacts with IpaB at the tip of the T3SS needle to form a translocon that inserts into lipid membranes (Blocker et al., 1999). An *ipaC*^−^ mutant has no haemolytic activity and is unable to escape the phagocytic vacuole (Bârzu et al., 1997). Given the role determined for IpaC in lysis of the invasion vacuole in epithelial cells (Osiecki et al., 2001; Du et al., 2016), it is very likely that IpaC also plays a role in this process in macrophages (Figure 1, step 3). *Salmonella*-induced vacuoles in macrophages have an acidic pH, and it was proposed that acidification may also be the cue to change IpaC function from cell entry to membrane lysis (De Geyter et al., 1997). If acidification is not the cue for vacuolar lysis, blocking acidification of endosomes using Bafilomycin-A_1_ should not prevent *Shigella* from exiting the vacuole.

#### IpaB: promotes macrophage pyroptosis

IpaB's hydrophobic region (310–430 amino acid residues) has 65% sequence identity to *Salmonella* invasive protein B (SipB), and is involved in invasion of the epithelium via translocon formation, phagosome escape, and induction of macrophage pyroptosis (Guichon et al., 2001). The binding site for the pro-pyroptotic and pro-inflammatory caspase-1, also known as interleukin-1β converting enzyme (ICE), is located at residues 311–401 within the hydrophobic region (Guichon et al., 2001). IpaB is localized at the bacterial surface and in discrete aggregates in the macrophage cytoplasm, which suggests that IpaB interacts with caspase-1 after vacuolar lysis rather than being injected into the cytoplasm from the vacuole (Thirumalai et al., 1997). The IpaB-ICE complex cleaves the precursors of pro-inflammatory cytokines IL-1β and IL-18 to produce mature IL-1β and IL-18, which are released in parallel to the induced pyroptosis (Figure 1, steps 4, 6). IpaB may also promote macrophage pyroptosis by allowing delivery of the T3SS the needle and rod proteins, MxiI and MxiH, into the cytosol. These bind the NAIP family of inflammasome receptors that trigger activation of caspase-1 (Yang et al., 2013; Suzuki et al., 2014b). Recent work also suggests that IpaD promotes macrophage apoptosis independent of caspase-1 but via host caspases accompanied by mitochondrial disruption (Arizmendi et al., 2016).

#### IpaH7.8: promotes macrophage pyroptosis

IpaH7.8 is part of the *ipaH* gene family found on the *Shigella* virulence plasmid. It was suggested that IpaH7.8 had a role in vacuolar lysis as an *ipaH7.8*^−^ mutant show reduced from escape the phagocytic vacuole (Fernandez-Prada et al., 2000). However, how this mutant strain (PWR700) was made is unclear, and its complementation was poor. Paetzold et al. (2007) then described how an *ipaH7.8*^−^ knockout was able to escape the phagosome. Therefore, IpaH7.8 has no role in vacuolar escape. Instead, the IpaH7.8 E3 ubiquitin ligase targets glomulin, an inhibitor of inflammasome activation, for ubiquitination leading to glomulin degradation. Macrophage-specific cell death (Figure 1, step 6) is then triggered through activated inflammasomes (Suzuki et al., 2014a).

### Modulation of adaptive immune system

#### LPS—serotype conversion and thymus-independent T-cell activation

**Figure 8:**
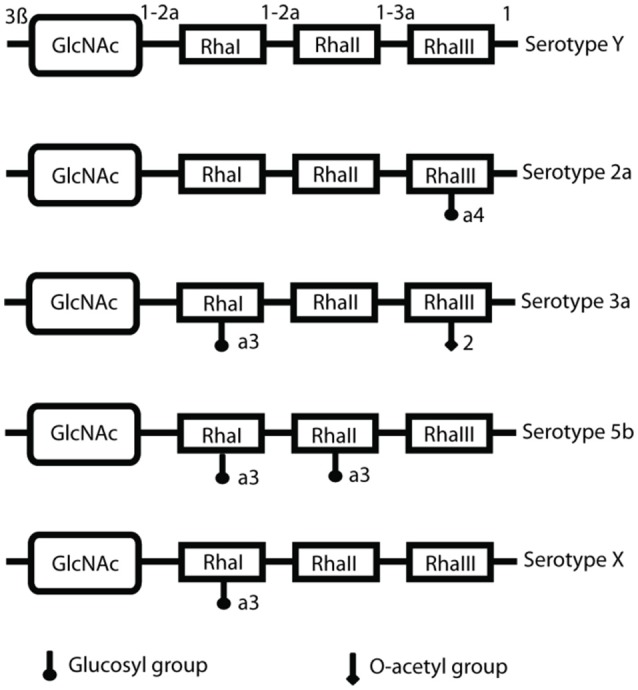
***S. flexneri* serotype composition and conversion**. LPS O-antigen modifications include glucosylation (the addition of glucosyl groups), mediated by the *gtr* operon and the addition of O-acetyl groups, achieved by the O-acetyltransferase protein encoded by the *oac* gene. This is the basis of serotype conversion, as *Shigella* begins with serotype Y and the basic O-antigen structure, and further modification produces different serotypes (Allison and Verma, 2000).

The acquisition of the SHI-O PAI means that *Shigella* is capable of modifying its LPS (Figure 8). As humoral immunity is serotype specific, and protection against re-infection is often unsuccessful because of the variety of *Shigella* serotypes. Furthermore, O-antigen of LPS is a carbohydrate, thymus-independent type 1 (TI-1) antigen, which activates B-cells in the absence of helper T-cells (Murphy, 2012). The lack of helper T-cell involvement means that these activated B-cells cannot undergo class switching or develop a memory B-cell response to protect against re-infection. At a low concentration of TI-1 antigens, such as when LPS molecules are released from damaged bacteria, the naïve B-cells are activated due to specific binding of their B-cell receptors to the antigen (i.e., O-antigen of LPS). This induces the production of O-antigen-specific antibodies, which are protective against *Shigella* infection, however they are not long-lasting and are overcome by serotype conversion. A high concentration of TI-1 antigens, like the O-antigen on LPS at the bacterial surface, leads to non-specific polyclonal activation of B cells and the production of non-specific and hence likely non-protective antibodies (Murphy, 2012). Thus, O-antigen behaves as an “immunological decoy” at more than one level, making it a particularly poor choice as a vaccine antigen.

#### IpgD: interferes with T-lymphocyte migration

**Figure 9:**
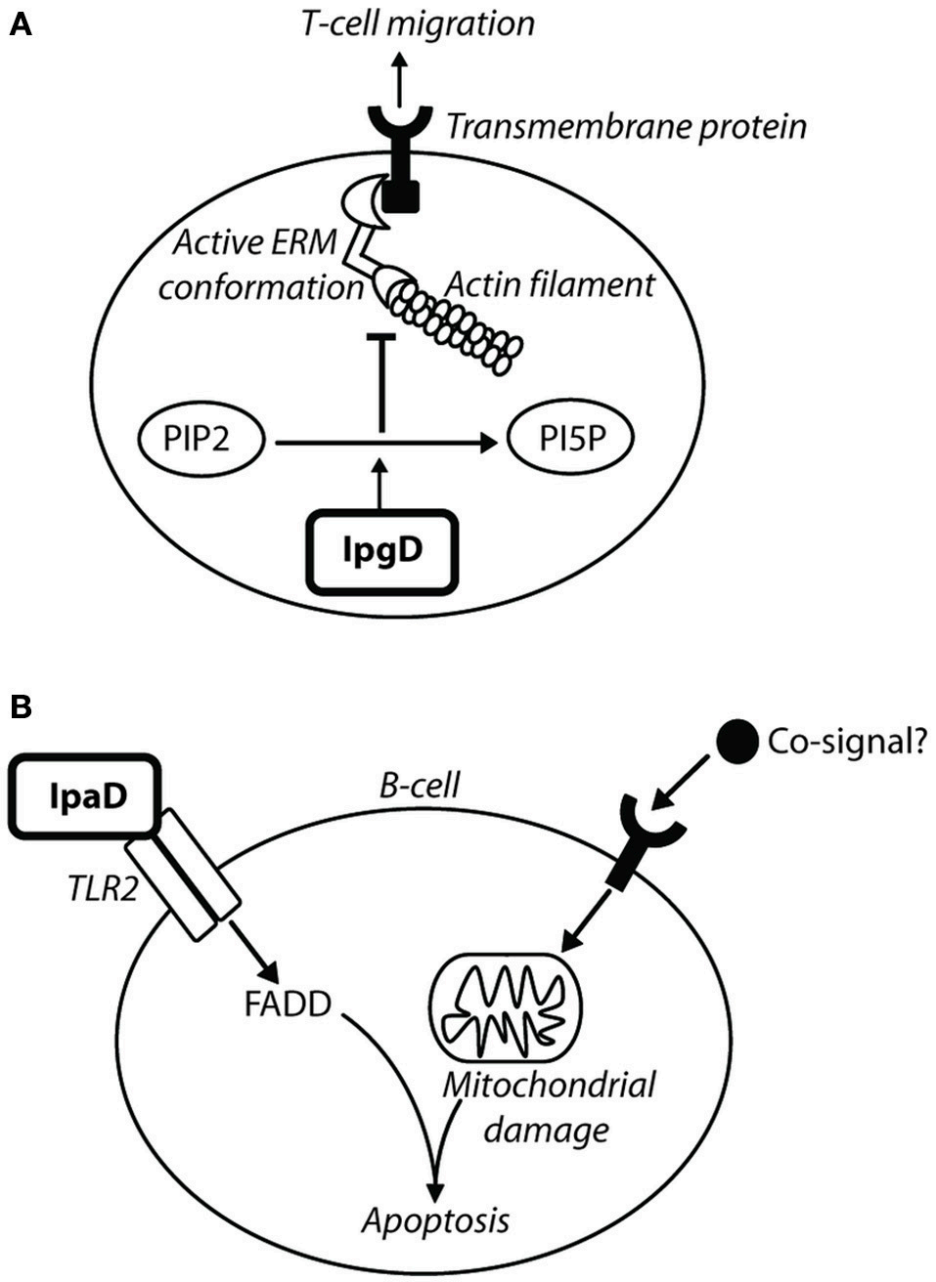
**IpgD inhibits T-cell migration and IpaD induces B-cell apoptosis. (A)** The PIP2 concentration at the plasma membrane is responsible for the dynamic interchange between active and inactive conformations of ezrin, radixin, and moesin (ERM) proteins (Konradt et al., 2011). ERM proteins are involved in cell-cortex organization of T-cells, and their active conformations localize at the membrane in response to chemokine stimulation to allow T-cell migration toward chemoattractants (Konradt et al., 2011). In the subcapsular sinus of the lymph node (Salgado-Pabón et al., 2013) *Shigella* can invade T-cells or inject IpgD via the T3SS into T-cells to reduce PtdIns(4,5)P_2_ concentration. This prevents cell polarisation induced by active ERM proteins and T-cell migration to sites of infection (Konradt et al., 2011). **(B)** Bacterial co-signals increase pro-apoptotic proteins, induce loss of mitochondrial membrane potential (MMP), and also upregulate *tlr2* mRNA, leading to increased TLR2 expression on the surface of the cell. IpaD signaling via the TLR2-1 heterodimer leads to up-regulation of FAS-associated death domain (FADD) protein, which ultimately induces B-cell apoptosis (Nothelfer et al., 2014).

IpgD is capable of interfering with T-cell migration by de-phosphorylating PtdIns(4,5)P_2_ (Figure 9). Wild-type *Shigella* causes a 50% decrease in T-cell migration toward a chemokine, such as CXCL12 (Konradt et al., 2011). Cells transfected with IpgD-GFP and incubated with CXCL12 had no ERM protein localization at the pole, preventing T-cell polarization and migration (Konradt et al., 2011). Mean velocity of T-cells was measured *in vivo*, with uninfected T-cells exhibiting 9 and 4 μm/min for wild-type *Shigella*-infected T-cells (Salgado-Pabón et al., 2013). This indicates that *Shigella* is capable of affecting T-cells in the context of infection, and that this ability is dependent on IpgD. *Shigella* may prevent T-cell migration and recruitment to areas of infection as they could be primed by CD1 antigen-presenting cells, which are involved in the presentation of lipid antigens, including LPS (Murphy, 2012). This could induce a specific antigen-response as priming of T-cells leads to stimulation of B-cells for isotype switching and immunological memory, therefore it is advantageous to prevent T-cell involvement in the adaptive immune response. Reducing migration of CD8^+^ cytotoxic T-cell may also slow clearance of infected cells from the epithelium.

#### IpaD: promotes B-lymphocyte apoptosis

To prevent an antibody response to the LPS, specific or non-specific, and production of immunological memory, *Shigella* also induces apoptosis in B-cells. This is mediated by IpaD in a manner independent of *Shigella* invasion and effector injection (Nothelfer et al., 2014). Incubation of B-cells with IpaD alone does not induce cell death, and it was deduced that bacterial co-signals work in conjunction with IpaD to mediate B-cell apoptosis (Figure 9) (Nothelfer et al., 2014). When anti-IpaD antibodies are applied to rectal biopsies of shigellosis patients, they are visualized within isolated lymphoid follicles and are contacting B-cells, suggesting that IpaD-induced B-cell apoptosis occurs *in vivo* (Nothelfer et al., 2014).

## Discussion

### How do the virulence factors work together?

#### Epithelial barrier destabilization and fluid secretion causes severe intestinal inflammation and diarrhoea

Once ingested, *S. flexneri 2a* delivers ShET1 in the jejunum to elicit fluid secretion (Fasano et al., 1997). Other *Shigella* species that harbor ShET2 require activation of the T3SS for its secretion and this most likely occurs later after contact with the colonic epithelium (Farfán et al., 2011). Pic and SigA, also harbored by *S. flexneri 2a* species, act within the lumen of the colon, on the apical side of the epithelium. Pic degrades the thick mucus layer by its mucinolytic activity to give *Shigella* better access to the epithelium (Gutierrez-Jimenez et al., 2008). SigA mediates enterotoxic activity similar to ShET1 (Al-Hasani et al., 2000) and may have cytopathic effects to initially destabilize the epithelial barrier (Al-Hasani et al., 2009). SepA causes fluid secretion in *S. flexneri 5a* (Benjelloun-Touimi et al., 1995). *Shigella* traverses the epithelium via M cells, and reaches the basolateral surface of the epithelium. Here the T3SS is activated, and *Shigella* can produce ShET2 to induce further fluid secretion (Nataro et al., 1995). Other T3SS effectors, including OspB (Zurawski et al., 2009; Ambrosi et al., 2014), OspC1 (Zurawski et al., 2006), and OspZ (Zurawski et al., 2008) (in *S. flexneri*) are secreted to phosphorylate and activate MAPK pathways. Consequently, increased apical secretion of the chemoattractant IL-8 causes PMN leukocytes to migrate across the epithelium in a basolateral to apical direction. This destabilizes the epithelial barrier, and the remaining bacteria at the apical surface can access the basolateral surface. *Shigella* that are phagocytosed by macrophages mediate pyroptosis via IpaB, T3SS components and probably LPS, which bind and activates caspase-1 (Thirumalai et al., 1997), and IpaH7.8 E3 ubiquitin ligase, which targets glomulin, an inflammasome inhibitor, for degradation (Suzuki et al., 2014a). Pyroptosis leads to release of pro-inflammatory cytokines IL-1β and IL-18, which further recruit PMN leukocytes and increase inflammation. All of these virulence determinants and effectors are therefore involved in the characteristic inflammation seen in shigellosis, including the watery diarrhea seen in the early stages. In *S. flexneri* 2a infection, mucus and blood may also be present due to the mucin secretagogue activity of Pic, and the cytopathic effects of SigA. Shiga toxin, found only in *S. dysenteriae*, may cause severe bloody dysentery by damaging the vascular endothelium of the colon (Fontaine et al., 1988).

#### Adhesion and entry to epithelium causes an epithelial genotoxic stress response

At the basolateral surface, glucosylation of the LPS is induced by an unknown trigger, and allows the T3SS to access the epithelium for activation (West et al., 2005). IpaB at the tip of the T3SS needle complex may bind to CD44 lipid microdomains to maintain contact of the T3SS with the epithelial cell (Skoudy et al., 2000), and IcsA increases polar adhesion (Brotcke Zumsteg et al., 2014). IpaB is inserted into the epithelial membrane, activating IpaD and leading to recruitment of IpaC to the host membrane to form the translocon pore (Blocker et al., 1999). IpaD activation also signals for the T3SS to facilitate early effector translocation through the T3SS needle and into the epithelial cell (Roehrich-Doenitz et al., 2013). IpaC, as one of the immediate effectors accessing the epithelial cell, mediates actin polymerization, either independently or indirectly via Cdc42 and Rac1 (Tran Van Nhieu et al., 1999; Mounier et al., 2009). This initiates membrane ruffles and entry foci, further promoted by IpgB1 and IpgB2, which remodel actin and the host cytoskeleton via their GEF activity (Huang et al., 2009). IpaA, in conjunction with host proteins, facilitates actin depolymerization, interfering with focal adhesions and preventing uncontrolled actin polymerization by IpaC (Tran Van Nhieu et al., 1997; Bourdet-Sicard et al., 1999). IpgD converts PtdIns(4,5)P_2_ to PtdIns(5)P, which reduces the membrane tether force and further stimulates membrane ruffle formation (Niebuhr et al., 2002). Together these effectors mediate the trigger mechanism for *Shigella* uptake into non-phagocytic epithelial cells. Upon enclosure in the primary entry vacuole, *Shigella* mediates membrane lysis. This involves primarily IpaB and/or IpaC in the translocon pore (Osiecki et al., 2001; Du et al., 2016), and as accessory processes IpgD-mediated recruitment of Rab11 to macropinosomes which make contact with the entry vacuole (Weiner et al., 2016), and/or VirA preventing growth of the vacuole membrane (Mellouk et al., 2014).

#### Intercellular spread and immune evasion causes erratic epithelial destruction and prevention of immunological memory

Once inside the epithelial cytoplasm, *Shigella* uses VirA and IpaJ to inactivate mainly Rab1 and ARF6, respectively. This halts ER-to-Golgi traffic, preventing autophagic membrane formation and inducing Golgi fragmentation (Dong et al., 2012; Burnaevskiy et al., 2013, 2015). *Shigella* further prevents autophagy using IcsB to mask the Atg5 binding site on IcsA (Ogawa et al., 2005). Unipolar localization of IcsA at the *Shigella* old pole, possibly achieved by LPS and IcsP, is crucial for efficient unidirectional movement (Monack and Theriot, 2001) and allows *Shigella* to move through the epithelial cytoplasm until it makes contact with the inner surface of the plasma membrane. It then protrudes into the adjacent epithelial cell, forming a secondary entry vacuole consisting of a double membrane. This is subsequently lysed by an unknown mechanism to allow access into the adjacent epithelial cell cytoplasm. Remaining within the epithelial layer is important for renewing the *Shigella* replication niche and also evading immune detection. Evasion of the immune response is achieved by T3SS effectors that inhibit activation pathways of NFκB, including OspG (Kim et al., 2005), OspI (Sanada et al., 2012), OspZ (Newton et al., 2010), IpaH9.8 (Ashida et al., 2010), and IpaH0722 (Ashida et al., 2013). Degradation of the pro-apoptotic factor p53 is achieved by IpgD (Mayo and Donner, 2001). Inhibition of the innate immune response also prevents development of an adaptive immune response. The movement of *Shigella* through the epithelium and the subsequent necrotic epithelial cell death is the cause of the colonic destruction and abdominal pain in shigellosis patients, contributing to prevention of fluid absorption and dysentery containing blood.

### Virulence determinants in disease-causing species

*S. flexneri* has a relatively stable genome, and acquired the virulence plasmid early in its evolution. *S. flexneri* is capable of persisting in water for several months, similarly to *Vibrio cholera* (Faruque et al., 2002). This may explain its epidemiological prevalence, as access to human hosts for months at a time could facilitate the endemics seen in countries with poor water sanitation. Speculatively, it may cause the most disease as *S. flexneri* 2a harbors the SHI-1 PAI, which encodes Pic, SigA, and ShET1. Pic may confer an advantage for scavenging nutrients, therefore other strains may have a metabolic disadvantage when compared to *S. flexneri* (Henderson et al., 1999; Harrington et al., 2009). The importance of Pic and ShET1 in pathogenesis has also been highlighted by Kotloff et al. (2004). The deletion of *pic* and *set1AB*, in addition to *ospD3*, in a guanine autotrophic (*guaAB*^−^) background produced an increasingly attenuated vaccine, with none of the 14 volunteers developing diarrhea (Kotloff et al., 2004). The truncated OspZ found in *S. flexneri* has a pro-inflammatory role, compared to its anti-inflammatory in the remaining sub-species, which may confer an inflammatory advantage for initial establishment of infection (Zurawski et al., 2008).

*S. sonnei* causes endemics in industrialized countries. Its emergence was defined by its acquisition of the pINVB plasmid, which harbors genes for the *Plesiomonas shigelloides*-related serotype 17 O-antigen (Shepherd et al., 2000). *P. shigelloides*, similarly to *S. flexneri*, can contaminate and persist in water sources. Infection with *P. shigelloides* from contaminated water may protect human hosts from established *S. sonnei* infection due to immunological memory and cross-reactive antibodies to the identical O-antigen (Sack et al., 1994). This hypothesis could also explain why an improvement in water sanitation causes a decrease in *S. flexneri* infection but an increase in *S. sonnei* infections, as there is no previous infection with *P. shigelloides* from contaminated water to induce a protective immunological memory response. Direct transmission of *S. sonnei* in schools and care settings is also capable of maintaining endemics in communities, without the requirement of an environmental reservoir.

*S. dysenteriae* infection is the most severe, which has been linked to the effects of the Shiga toxin harbored by *S. dysenteriae* type 1. There is no known natural reservoir for *S. dysenteriae*, and it causes sporadic epidemics linked to poor hygiene and overcrowding. The Shiga toxin does not play a role in the intracellular infection, but can stimulate the recruitment of PMN leukocytes and damages the colonic vascular endothelium, leading to the characteristic blood-containing dysentery (Fontaine et al., 1988). It has been suggested that, like *Salmonella enterica* serovar Typhi, *S. dysenteriae* can be maintained and transmitted within a community by an asymptomatic carrier. Long term carriers with attenuated symptoms have been previously reported (Levine et al., 1973; Clements et al., 1988), and this may explain the epidemiology of *S. dysenteriae* epidemics, which disappear to then reappear years later, and are also transferred intercontinentally (Rohmer et al., 2014).

*S. boydii* causes the least burden of disease worldwide. It represents 1–2% of *Shigella* isolated, and is mostly confined to the Indian subcontinent. In 2000, *S. boydii* serotype 20 was discovered. Its transmission was linked to travel in Mexico (Kalluir et al., 2004) and it was the most frequent agent of *S. boydii* infection in Canada (Woodward et al., 2005). However, *S. boydii* still only caused 1% of *Shigella* infections in the United States compared to the 77% of infections caused by *S. sonnei* (Kalluir et al., 2004). Due to the diversity of *S. boydii* serotypes, and its lack of clinical relevance there is little known about its virulence determinants and why it causes less disease than *S. flexneri, S. sonnei*, and *S. dysenteriae*.

### Effector interplay

#### Multiple effectors for one target pathway

Effector-Triggered Immune Pathology (ETIP) is an advanced model of both the Guard Hypothesis and Effector-Triggered Immunity (ETI). If the host is “resistant” and encodes the guardee protein, it is capable of recognizing the damage mediated by *Shigella* effectors and this induces an efficient innate immune response. *Shigella* can use this to its advantage, increasing intestinal inflammation which is required for infection establishment and transmission by diarrhea (Stuart et al., 2013). However, this inflammation eventually leads to the clearance of infection, therefore *Shigella* harbors a large repertoire of anti-inflammatory mechanisms for down-regulation of ETIP. The host is not able to “guard” all the steps/signaling pathways involved in NFκB activation, therefore multiple effectors with different biochemical functions are capable of efficiently interfering with these pathways and dampening the host immune response. Similarly, VirA and IpaJ act to fragment the Golgi via exertion of differing biochemical activities on different small GTP binding proteins involved in its maintenance.

#### More than one effector with a similar biochemical function

IpgB1 and IpgB2 are similar in their WxxxE motif and GEF activity, but they have non-overlapping substrates. Only a dual *ipgB1*^−^*ipgB2*^−^ knockout had a negative Sereny test, indicating that they also have similar but non-overlapping functions (Hachani et al., 2007). Lack of redundancy was also confirmed, as an *ipgB2*^−^ knockout has the same phenotype as the wild-type strain, however the *ipgB1*^−^ knockout had an increase in inflammation compared to the wild-type strain. This may link to the guard hypothesis, whereby IpgB1 prevents detection of IpgB2 by the host guardee proteins, perhaps via its own non-overlapping enzyme activity, to prevent an excessive inflammatory response.

#### One effector with multiple functions?

An effector characterized with multiple roles in pathogensis is normally due to a single function which mediates several effects. For example, the only characterized activity of IpgD is that it dephosphorylates phosphoinositides, decreasing levels of PtdIns(4,5)P_2_ and increasing levels of PtdIns(5)P. Changes in the intracellular concentrations of these phosphoinositides then produces effects directly, such as membrane ruffles (Niebuhr et al., 2002), or indirectly, such as vacuolar lysis (Mellouk et al., 2014), activation of Akt/PI3K signaling pathway (Pendaries et al., 2006), and interference with T-lymphocyte migration (Konradt et al., 2011). Therefore, IpgD has one biochemical activity, which mediates multiple effects on the host cell. This is also probably the case for IpaJ in Golgi fragmentation and STING activation and for OspB in modulating cell-to-cell spread via mTOR and ERK/MAPK signaling. Some factors, such as IcsA involved in adhesion and actin-tail formation, may genuinely have two very different biochemical functions, used at different points in the infectious cycle. OspE1/E2 seem involved in adhesion to host cells and stability of tight junctions. However, others may not. At its position at the T3SS needle tip, IpaB is involved in CD44 interactions (Skoudy et al., 2000), host cell sensing, translocon pore formation (Blocker et al., 1999), and directly or indirectly, and lysis of the single and double membrane entry vacuoles (High et al., 1992; Page et al., 1999). Once secreted, IpaB plays a role in macrophage pyroptosis (Guichon et al., 2001) and cell cycle arrest (Iwai et al., 2007). It is difficult to comprehend how IpaB could exert all these functions without possessing multiple biological activities, which remain unclear. However, some of these functions may have been misattributed to IpaB due to pleotropic effects stemming from its involvement in translocon formation, which is crucial for *Shigella* entry.

## Concluding remarks

The vast majority of the functional work presented here has been done on *S. flexneri*. Comparative analysis of the dominant *Shigella* genomes causing disease is required for further analysis of virulence determinants, such as any involved lysis of the single and double membrane entry vacuoles, which remain completely unclear, and of their epidemiological consequences. The importance of such genomic investigations has recently been highlighted (The et al., 2016). Identification of important and conserved effectors and other virulence determinants that cause disease will contribute to an overall understanding of infection, further illuminating species tropism and transmission and helping to create a pan-*Shigella* vaccine, which so far has been unsuccessful.

## Author contributions

EM planned, researched, wrote, and subsequently also formatted the manuscript for publication, assisted by discussions, analysis of published works and editing with AB. EM also generated Figures 5–9.

## Funding

AB is the recipient of an Investigator Award (WT104634AIA) from the UK Wellcome Trust. EM was an undergraduate student in the School of Cellular and Molecular Medicine at the University of Bristol, UK.

### Conflict of interest statement

The authors declare that the research was conducted in the absence of any commercial or financial relationships that could be construed as a potential conflict of interest.
